# Data-based modeling for hypoglycemia prediction: Importance, trends, and implications for clinical practice

**DOI:** 10.3389/fpubh.2023.1044059

**Published:** 2023-01-26

**Authors:** Liyin Zhang, Lin Yang, Zhiguang Zhou

**Affiliations:** National Clinical Research Center for Metabolic Diseases, Key Laboratory of Diabetes Immunology, Ministry of Education, Department of Metabolism and Endocrinology, The Second Xiangya Hospital of Central South University, Changsha, China

**Keywords:** diabetes mellitus, hypoglycemia, prediction, data-based algorithms or models, machine learning

## Abstract

**Background and objective:**

Hypoglycemia is a key barrier to achieving optimal glycemic control in people with diabetes, which has been proven to cause a set of deleterious outcomes, such as impaired cognition, increased cardiovascular disease, and mortality. Hypoglycemia prediction has come to play a role in diabetes management as big data analysis and machine learning (ML) approaches have become increasingly prevalent in recent years. As a result, a review is needed to summarize the existing prediction algorithms and models to guide better clinical practice in hypoglycemia prevention.

**Materials and methods:**

PubMed, EMBASE, and the Cochrane Library were searched for relevant studies published between 1 January 2015 and 8 December 2022. Five hypoglycemia prediction aspects were covered: real-time hypoglycemia, mild and severe hypoglycemia, nocturnal hypoglycemia, inpatient hypoglycemia, and other hypoglycemia (postprandial, exercise-related).

**Results:**

From the 5,042 records retrieved, we included 79 studies in our analysis. Two major categories of prediction models are identified by an overview of the chosen studies: simple or logistic regression models based on clinical data and data-based ML models (continuous glucose monitoring data is most commonly used). Models utilizing clinical data have identified a variety of risk factors that can lead to hypoglycemic events. Data-driven models based on various techniques such as neural networks, autoregressive, ensemble learning, supervised learning, and mathematical formulas have also revealed suggestive features in cases of hypoglycemia prediction.

**Conclusion:**

In this study, we looked deep into the currently established hypoglycemia prediction models and identified hypoglycemia risk factors from various perspectives, which may provide readers with a better understanding of future trends in this topic.

## 1. Introduction

Diabetes mellitus is a chronic disease characterized by high blood glucose caused by the inability to produce or effectively use insulin. Maintaining blood glucose within the normal range may help prevent or delay the development of diabetic microvascular or macrovascular complications (1–4). However, intensive glycemic control increases the frequency of hypoglycemia while decreasing the risk of long-term complications (5). Hypoglycemia is usually defined as a blood glucose concentration of < 70 mg/dL. Counterregulatory response and impaired cognitive function are two physical changes brought on by hypoglycemia (6–8). In addition to impaired behavioral functions such as fear of hypoglycemia (9), depression (10), and dyskinesia (11, 12), hypoglycemia can also result in fatal cardiovascular events (13).

Clinically, hypoglycemia can be classified as mild (MH) or severe (SH) depending on whether third-party assistance is required during hypoglycemia episodes or if there is a loss of consciousness (14). The frequencies of MH and SH episodes in patients with type 1 diabetes (T1D) have been estimated to be 1.6 and 0.029 episodes per person-week, respectively (15), and the incidence of SH was approximately 0.44 episodes per person-year in insulin-treated patients with type 2 diabetes (T2D) (16). Furthermore, hypoglycemia is more common in patients with diabetes who are hospitalized (17, 18). SH is extremely dangerous and is more likely to occur in patients on long-term insulin therapy (5). The counterregulatory response is critical in preventing MH from progressing to SH. However, in individuals with recurrent hypoglycemia, the frequency of hypoglycemic warning symptoms tends to decrease gradually, leading to impaired awareness of hypoglycemia (IAH) and the occurrence of asymptomatic hypoglycemia (19, 20), and IAH, in turn, increases the risk of SH (21, 22), eventually forming a vicious cycle.

Clinical attention is also given to nocturnal hypoglycemia, exercise-related hypoglycemia, and inpatient hypoglycemia. In T1D patients known as “fragile diabetes”, nocturnal hypoglycemia (NH) accounted for 55% of hypoglycemia when a blood glucose concentration < 54 mg/dL (23–25), and this proportion increased to 75% in pediatric patients (26). Patients may be unable to detect episodes of NH in time while sleeping, which may predispose them to IAH in the long run or even result in “dead-in-bed syndrome”. Aside from the benefits of physical activity, such as improving cardiopulmonary adaptability, blood lipid levels, and lowering the risk of long-term cardiovascular events in patients with diabetes (27–29), it may also cause post-exercise hypoglycemia due to increased insulin sensitivity in the short term (30), resulting in avoidance of exercise due to fear of hypoglycemia. Furthermore, symptoms of early hypoglycemia in T1D patients may be masked by physical activity, which may increase the frequency of SH in this population (31). The frequent occurrence of iatrogenic hypoglycemia in hospitalized patients is also not negligible. A systematic review has shown that intensive glucose control increases the risk of inpatient hypoglycemia (32). It was estimated that patients hospitalized for diabetes or hyperglycemia experience an average of two hypoglycemic events per week, with the majority occurring during the night (33). Worse still, inpatient hypoglycemia was associated with a variety of negative outcomes, including increased mortality and serious cardiovascular events (13).

Hypoglycemia prediction is crucial in clinical practice. Many studies have emerged in the last decade that used conventional approaches based on physiological and clinical parameters to predict hypoglycemia (34). These approaches were typically trained using retrospectively collected demographic data, laboratory test results, glucose-lowering agents, and other indicators that may be associated with hypoglycemia obtained from electronic health records in a certain period (35, 36). And the most commonly used statistics were linear regression, logistic regression, Cox hazards regression, and other operations tailored to the problem at hand.

Machine learning (ML) advances, on the other hand, and the need for more accurate hypoglycemia prediction has resulted in data-driven predictive models. Benefiting from the rapid development of continuous glucose monitoring (CGM), the risk of hypoglycemia could be simply predicted by specific parameter calculations such as low blood glucose index (LBGI) (37, 38). Complex CGM-based hypoglycemia predictions were used to develop and improve the artificial pancreas (AP) algorithm, which alerts users when blood glucose levels drop or hypoglycemia is imminent (39). Data-driven algorithms and ML models improve hypoglycemia prediction (40). CGM glucose data are frequently used for ML model establishment due to their continuity and bulkiness.

According to the prediction horizon (PH), such models can be divided into short-term (< 180 min), mid-term (180 min to 24 h), and long-term (several days, months, or even years). Undoubtedly, the prediction efficiency of CGM-based models would decrease with the extension of PH. Since glucose autocorrelation usually disappears after 30 min (41), and 30 min is the minimum time interval for effective patient intervention to prevent accidents, current CGM-based hypoglycemia prediction models set PH at 30 min or above. In brief, current hypoglycemia prediction models are mostly based on clinical parameters, CGM data, or a combination of both. The predictive accuracy varied with the study population, outcome definition, PH definition, modeling technique and model training and validation approaches (42). As for clinical data-based models, large sample size and sufficient data processing are frequently required to ensure the accuracy and reliability. Such models typically collect clinical hypoglycemic events for risk stratifying and further hypoglycemia-associated feature selection, followed by internal or external validation of model generalizability. The PH of such models tends to be broad, ranging from predicting short-term hypoglycemic events like inpatient hypoglycemia to long-term hypoglycemic events like SH events months later. It is important to note that a shorter PH may be more useful for prompt clinician intervention, whereas a larger PH increases the prevalence of an outcome and consequently model performance, but may be less useful as a decision support tool (42).

Felizardo et al. (34) conducted a systematic review of data-based algorithms and models in blood glucose prediction, which could be considered an extension of the work of Oviedo et al. (43). They only looked at ML approaches to prediction, and the relevant literature was from before June 2020. Nonetheless, no study comparing conventional and data-based hypoglycemia risk prediction has been discussed and compared from a more holistic clinical perspective to our knowledge. In this study, we investigated the currently established hypoglycemia prediction models and identified hypoglycemia risk factors from various perspectives, which may provide patients and clinicians with a better understanding of future trends in this topic. Our review will be discussed from three perspectives: (1) an overview of currently established hypoglycemia prediction models; (2) dividing the selected studies into five hypoglycemia prediction parts: real-time hypoglycemia, MH/SH, NH, inpatient hypoglycemia, and other hypoglycemia (postprandial, exercise-related), explored hypoglycemia risk factors and illustrated prediction approaches from clinical and ML perspectives, respectively; (3) a comprehensive evaluation and comparison of current hypoglycemia prediction models, with clinical implications and insights into future trends.

## 2. Methods

### 2.1. Search strategy

**Figure 1:**
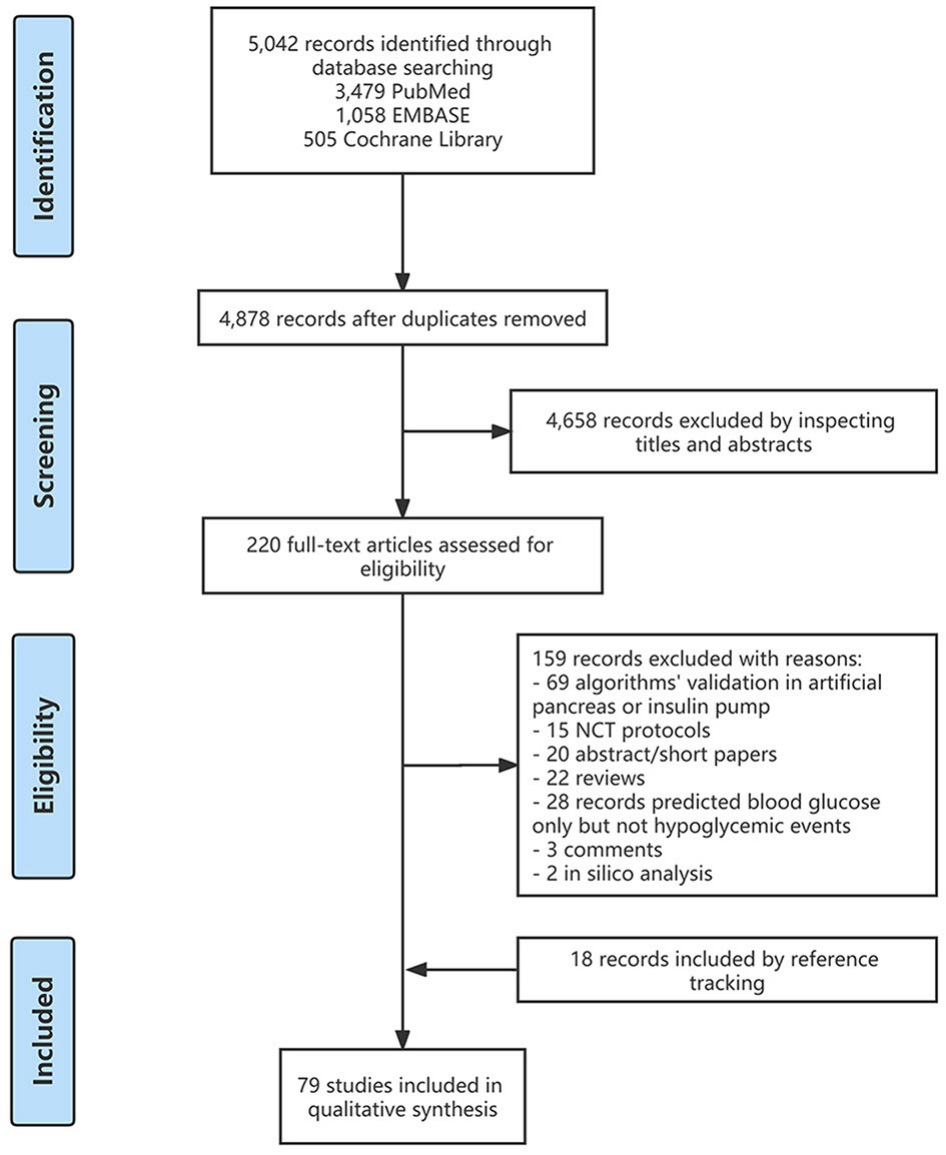
Flow diagram of the selection process.

We searched the PubMed, EMBASE, and Cochrane Library databases for relevant literature from 1 January 2015 to 8 December 2022. The search keywords were “diabetes”, “hypoglycemia prediction”, “hypoglycemia warning”, “hypoglycemia detection” and “hypoglycemia estimation”. A total of 5,042 records were found, with 61 of them being relevant to our topic. In addition, we manually added 18 records related to the included records after full-text reading and reference tracking. Figure 1 depicts the flow diagram of detailed literature inclusion.

### 2.2. Inclusion and exclusion criteria

To include as many relevant records as possible to increase the reliability of our review, we included the records which met the following conditions: (1) the study population were patients with abnormal glucose tolerance or diagnosed with diabetes, regardless of the age of the patients and the type of diabetes; (2) the original data used for the analysis of factors related to hypoglycemia in the records and the establishment of predictive algorithms or models were the patient basic data, medication regimen, laboratory test results and blood glucose measurement results (self-monitoring of blood glucose [SMBG] or CGM results) or other indices retrieved from the real-world studies, clinical trials or cohort studies; (3) the main results of the record were detailed and were exact correlations, algorithms or models related to hypoglycemia prediction. We excluded the following types of records: (1) the research topic was the algorithm proposal or improvement of hypoglycemia warning in AP or CGM products; (2) NCT protocols; (3) abstract/short papers; (4) reviews; (5) blood glucose concentration predictions only but not hypoglycemic events; (6) comments; (7) *in silico* study.

### 2.3. Data extraction and quality assessment

Data were extracted from the full text and supplementary information of eligible records according to pre-established literature classification criteria. For each study, the following data were carefully extracted: first author, year of publication, use of database features (study population, sample size, type of input clinical data for modeling, source of input glucose data for modeling, modeling approach), type of hypoglycemia prediction (real-time hypoglycemia, MH/SH, NH, inpatient hypoglycemia, other hypoglycemia [postprandial, exercise-related]). Then, the algorithms or models used, the performance, as well as the significance of model performance metrics were tabulated in detail according to the type of hypoglycemia prediction.

### 2.4. Research emphasis

1) An overview of the currently established prediction model of hypoglycemia;
2) divided the selected studies into five hypoglycemia prediction parts: real-time hypoglycemia, MH/SH, NH, inpatient hypoglycemia, and other hypoglycemia (postprandial, exercise-related), explored the risk factors of hypoglycemia and illustrated prediction approaches from the perspective of clinical and machine learning, respectively;
3) a comprehensive evaluation and comparison of current hypoglycemia prediction models, extraction of clinical implications, and insights into the future trends in this topic.

## 3. Results

### 3.1. Eligible studies

**Figure 2:**
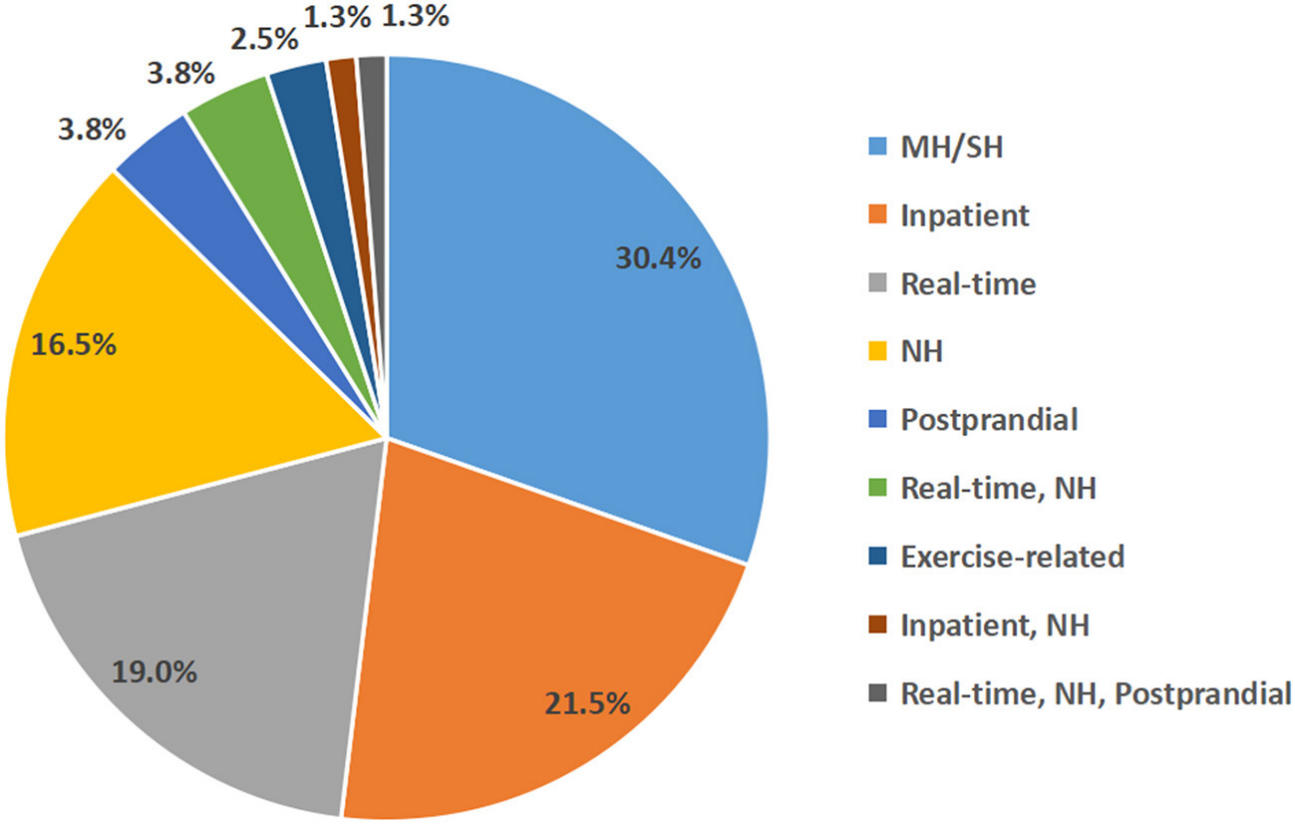
The prediction type and proportion of selected studies.

Of the 5,042 records obtained after the initial search, 220 records remained after the primary screening of titles and abstracts and were assessed for eligibility, and 159 records were excluded after full-text review, as follows: (1) 69 records were related to the validation of algorithms in the AP or insulin pump; (2) 20 records were without full text; (3) 15 records were protocols for randomized controlled trials; (4) 22 records were reviews; (5) 28 records only predicted blood glucose concentrations but not hypoglycemic events; (6) three records were comment, and (7) two records were *in silico* studies (Figure 1). Furthermore, we added 18 relevant studies through reference tracking. As a result, the total number of studies included was 79: 15 studies on real-time hypoglycemia prediction (19.0%), 24 studies on MH/SH prediction (30.4%), 13 studies on NH prediction (16.5%), 17 studies on inpatient hypoglycemia prediction (21.5%), two studies on exercise-related hypoglycemia prediction (2.5%), and three studies on postprandial hypoglycemia prediction (3.8%), three studies on real-time and NH prediction (3.8%), 1 study on real-time and inpatient hypoglycemia prediction (1.3%) and 1 study on real-time, NH and postprandial hypoglycemia prediction (1.3%) (Figure 2). Details of all included articles are summarized in Table 1.

**Table 1:** An integrated analysis of the selected studies.

**References**	**Diabetes**	**Sample**	**Clinical inputs**	**Glucose inputs**	**Algorithms**	**PH**	**Outcome definition**	**Validation approach**	**Model performance**	**Prediction type**
Gerstein et al. (44)	IFG/IGT, T2D	12,537 participants, ≥ 50 years	Demographics, Lab data, GLA, comorbidities		Cox regression		Hypoglycemia ≤ 54 mg/dL, ≤ 36 mg/dL			MH/SH
Bordier et al. (45)	T2D	987 participants, ≥ 70 years	Demographics, Lab data, GLA, comorbidities, COM, mental questionnaires results		LR		Hypoglycemia < 60 mg/dL			MH/SH
Cariou et al. (46)	T1D, T2D	4,424 participants, ≥ 18 years	Demographics, Lab data, GLA, comorbidities, COM, previous HYPO episodes	SMBG	LR		Hypoglycemia < 70 mg/dL			MH/SH
Cichosz et al. (47)	T1D	21 participants, 58 years	Heart rate variability	CGM	Pattern classification	20 min	One single SG < 70 mg/dL (5 min)	10-fold cross validation	AUC = 0.96, Se = 100%, Sp = 91%	Real-time
Ganz et al. (48)	T2D	7,235 participants, ≥ 18 years	Demographics, GLA, previous SH/healthcare/medication		LR		Hypoglycemia ≤ 40 mg/dL			MH/SH
Inzucchi et al. (49)	T2D	1,699 participants, 59.4 years	CGM parameters	CGM	Correlation					MH/SH
Samuel et al. (50)	T2D	NR	GLA, HbA1c, diabetes duration, GFR, BMI		Mathematical model		Hypoglycemia < 70 mg/dL, 50 mg/dL	External		MH/SH
Sonoda et al. (51)	T1D, T2D	123 participants, 65.9 years	Social factors, lifestyle factors, Lab data, GLA		LR		Hypoglycemia < 70 mg/dL, ≤ 49 mg/dL			MH/SH
Sudharsan et al. (52)	T2D	163 participants, 52.8 years	Medication	SMBG	RF, SVM, k-nn, naïve Bayes	24 h, the 8th day	Hypoglycemia < 70 mg/dL	Cross validation	24 h: Se = 91.7%, Sp = 69.5% 8th day: Se = 90.4%, Sp = 91.1%	MH/SH
Ling et al. (53)	T1D	16 participants, 14.6 years	ECG	SMBG	ELM-based NN	3 min	Hypoglycemia < 60 mg/dL	Random subsampling	Se = 78.0%, Sp = 60.0%	NH
Sampath et al. (54)	T1D	34 participants, 18–65 years	CGM parameters	CGM	Aggregating ranking	Nighttime	Hypoglycemia < 70 mg/dL	External	Se = 77.0%, Sp = 83.4%	NH
Tkachenko et al. (55)	T1D	34 participants, 18–65 years	CGM raw data and parameters	CGM	Aggregating ranking	Nighttime	Hypoglycemia < 70 mg/dL	Random subsampling	Se = 73.4%, Sp = 87.8%	NH
Klimontov et al. (56)	T2D	83 participants, 65–80 years	CGM parameters	CGM	LR	Nighttime	Hypoglycemia ≤ 70 mg/dL		Acc = 75.6%, Se = 84.0%, Sp = 62.1%	NH
Karter et al. (57)	T2D	206,435 participants, ≥ 21 years	Demographics, insulin/ SU use, history of HYPO-related utilization, prior year ED use		Recursive partitioning	1 year	Hypoglycemia-related ED or hospital use	Internal, external	Internal validation: c-statistic = 0.83, external validation 1/2: c-statistic = 0.81/0.79	MH/SH
Schroeder et al. (58)	T1D, T2D	70,438 participants, 59.8 years	Demographics, diabetes type, Lab data, GLA, comorbidities, COM, previous HYPO events		Cox regression	6 months	Hypoglycemia < 70 mg/dL	External	External validation 1/2: c-statistic = 0.80/0.84	MH/SH
Stuart et al. (59)	T1D, T2D	9,584 participants, > 16 years	Demographics, Lab data, comorbidity score, GLA, previous type of admission		LR	Hospital stay	Hypoglycemia < 70 mg/dL	Bootstrapping	AUC = 0.733	Inpatient
Ena et al. (60)	DM	1,400 participants	Demographics, Lab data, comorbidities, GLA		LR	Hospital stay	Hypoglycemia < 70 mg/dL	External	Validation: AUC = 0.71	Inpatient
Sakurai et al. (61)	T2D	50 participants, 64 years	Demographics, Lab data	SMBG, CGM	Mathematical formula		Lowest nocturnal blood glucose		R^2^ = 0.90	NH
Chow et al. (62)	T2D	10,251 participants, 62.8 years	Demographics, GLA, comorbidities, previous HYPO events, other medication		Cox regression	5 years	Hypoglycemia ≤ 50 mg/dL	5-fold cross validation	Validation: c-statistic = 0.782	MH/SH
Torimoto et al. (63)	T2D	294 participants, 62.2 years	Demographics, COM, Lab data, GLA, CGM parameters	CGM	LR		One single SG < 70 mg/dL (5 min)			MH/SH
Han et al. (64)	T2D	1,676,885 participants, 57.9 years	Demographics, GLA, current smoking, exercise, insulin, comorbidities, fasting glucose levels, previous HYPO events		Cox regression	1 year	SH events identified by ICD-10 codes	Bootstrapping	Validation: c-statistic = 0.866, Se = 80.2%, Sp = 79.7%	MH/SH
Elvebakk et al. (65)	T1D	20 participants, 41.1 years	Sweating, skin temperature, ECG, counter-regulatory hormones, symptoms of HYPO		ROC analysis					MH/SH
Winterstein et al. (66)	DM	21,840 participants, > 18 years	Demographics, GLA, Lab data, oral intake related, service location related, comorbidities	SMBG	LR	24 h	Hypoglycemia < 50 mg/dL not followed by glucose value > 80 mg/dL within 10 min	Bootstrapping	On day 3–5: c-statistic = 0.877	Inpatient
Mathioudakis et al. (67)	DM	19,262 participants, 61.3 years	Demographics, diagnoses, insulin, comorbidities, Lab data, medications, diet order, steroid use, BG readings		LR	24 h	Hypoglycemia ≤ 70 mg/dL, < 54 mg/dL	Internal	≤ 70 mg/dL: c-statistic = 0.77; < 54 mg/dL: c-statistic = 0.80	Inpatient
Cichosz et al. (68)	T1D	56 participants, 68.7 years	Heart rate variability	CGM	Pattern classification	20 min	One single SG < 70 mg/dL (5 min)	Internal	AUC = 0.95	Real-time
Elvebakk et al. (69)	T1D	20 participants, 41.1 years	ECG, near-infrared and bioimpedance spectroscopy	SMBG	Probabilistic model					MH/SH
Jaggers et al. (70)	T1D	10 participants, 13–17 years	Physical activity intensity	CGM	LR		Two consecutive SG < 70 mg/dL (10 min)			NH
Li et al. (71)	DM	38,780 participants, 57 years	Demographics, Lab data, previous HYPO events, GLA, comorbidities, COM, insurance		LR, CART, RF	2 years	Hypoglycemia < 70 mg/dL	10-fold cross validation	AUC = 0.89 for LR model, AUC = 0.88 for CART model, AUC = 0/90 for RF model	MH/SH
Oviedo et al. (72)	T1D	10 participants, 41 years	Insulin, carbohydrate intake, BG level at mealtime	CGM	naive Bayes, AdaBoost, SVM, ANN	6 h	Three consecutive SG < 70 or 54 mg/dL (15 min)	5-fold cross validation	< 70 mg/dL: Se = 49.0%, Sp = 74.0%; < 54 mg/dL: Se = 51.0%, Sp = 74.0%	Postprandial
Oviedo et al. (73)	T1D	10 participants, 41 years	Insulin, carbohydrate intake	CGM	SVM	6 h	Three consecutive SG < 70 or 54 mg/dL (15 min)	5-fold cross validation	< 70 mg/dL: Se = 71.0%, Sp = 79.0%; < 54 mg/dL: Se = 77.0%, Sp = 81.0%	Postprandial
Shah et al. (74)	DM	585 participants, 69.9 years	Demographics, previous HYPO events, Lab data, GLA, CKD status		LR	Hospital stay	Hypoglycemia ≤ 70 mg/dL	External	Validation: c-statistic = 0.642, Se = 77.0%, Sp = 28.0%	Inpatient
Tronstad et al. (75)	T1D	20 participants, 18-60 years	Near-infrared, bioimpedance, skin temperature		PLS, ANN		Hypoglycemia < 72 mg/dL			MH/SH
Vu et al. (76)	T1D	9,800 participants, 45.3 years		CGM	RF	3 h, 6 h	Three consecutive SG < 70 mg/dL (15 min)	10-fold cross validation	3h: AUC = 0.90; 6 h: AUC = 0.84	NH
Reddy et al. (77)	T1D	43 participants, 33 years	Demographics, exercise, glucose, hormone features		DT, RF	During exercise	Hypoglycemia < 70 mg/dL	10-fold cross validaton	Acc = 86.67%, Se = 86.21%, Sp = 86.89%	During exercise
Yang et al. (78)	T1D, T2D	100 participants, 44.8 years		CGM	ARIMA	30 min	Three consecutive SG ≤ 70 mg/dL (9 min)		Se _T1D/T2D_ = 100.0/100.0%; FPR _T1D/T2D_ = 10.7/8.0%	Real-time
Gadaleta et al. (79)	T1D	89 participants		CGM	SVR	30 min	Hypoglycemia ≤ 70 mg/dL	Leave-one-out validation	Se = 75.0%, PPV = 51.0%	Real-time
Seo et al. (80)	T1D, T2D	104 participants, > 18 years		CGM	RF, SVM, k-nn, LR	30 min	One single SG ≤ 70 mg/dL (5 min)	5-fold cross validation	Se = 89.6%, Sp = 91.3%	Postprandial
Choi et al. (81)	DM	487 participants, 51.8 years	Demographics, GLA, Lab data, previous BG control		Description					Inpatient
Bertachi et al. (82)	T1D	10 participants, > 18 years	CGM raw data, meals, insulin, heart rate signal, steps, calories burned, sleep period	SMBG, CGM	MLP, SVM	Nighttime	One single SG < 70 mg/dL (15 min)	5-fold cross validation	MLP: Acc = 77.38%, Se = 69.52%, Sp = 78.98%; Acc = 80.77%, Se = 78.75%, Sp = 82.15%	Real-time, NH
Elhadd et al. (83)	T2D	13 participants, 51 years	Demographics, Lab data, physical activity, medication	CGM	LR, RF, XGBoost, SVM	During Ramadan			Acc = 27.9%	MH/SH
Hu et al. (84)	T2D	257 participants	Demographics, Lab data, COM, comorbidities		LR	Hospital stay	Hypoglycemia ≤ 70 mg/dL	Bootstrapping	AUC = 0.664	Inpatient
Jensen et al. (85)	T1D	463 participants, 43 years	Demographics, meal, insulin	CGM	LDA	Nighttime	Three consecutive SG ≤ 54 mg/dL (15 min)	5-fold cross validation	AUC = 0.79, Se = 75.0%, Sp = 70.0%	NH
Khanimov et al. (86)	DM	1,342 participants, 75 years	Nutrition risk screening 2002, admission serum albumin		Cox regression	Hospital stay	Hypoglycemia ≤ 70 mg/dL		Acc = 49.0%, Se = 70.0%, Sp = 46.0%	Inpatient
Khanimov et al. (87)	DM	7,718 participants, 71.8 years	Admission serum albumin, blood osmolarity, Charlson Comorbidity Index		Cox regression	Hospital stay	Hypoglycemia ≤ 70 mg/dL			Inpatient
Li et al. (88)	T1D, T2D	1,921 participants, 59 years		CGM	LR, SVM, RF, LSTM	30 min	Three consecutive SG ≤ 70 mg/dL (15 min)	Internal	Se = 92.05%, FPR = 7.69%	Real-time, NH
Ma et al. (89)	T2D	10,251 participants, 62.2 years	Demographics, Lab data, medications, physical exam findings, mental health results		MMTOP		Hypoglycemia < 50 mg/dL	10-fold cross validation	C-statistic = 0.77	MH/SH
Marcus et al. (90)	T1D	11 participants, 18-39 years		CGM	KRR	30 min	Hypoglycemia < 70 mg/dL	Hold-out validation	Se = 64.0%, FPR = 4.0%	Real-time
Misra-Hebert et al. (91)	T2D	1,876 participants, 64.7 years	Demographics, Lab data, comorbidities, GLA, previous HYPO events		LR	3 months	SH events identified by diagnosis code	Bootstrapping	AUC = 0.89, Se = 82.0%, Sp = 79.0%	MH/SH
Misra-Hebert et al. (92)	T2D	47,280 participants, 61.4 years	Demographics, Lab data, comorbidities, GLA, previous HYPO events, insurance type		Cox regression		SH events identified by diagnosis code			MH/SH
Mosquera-Lopez et al. (93)	T1D	124 (31 years), 10 (34 years) participants	Insulin, carbohydrate intake	CGM	SVR	Nighttime	One single SG < 70 mg/dL	External	AUC = 0.86, Se = 94.1%, Sp = 72.0%	NH
Tran-Duy et al. (94)	T1D	27,841 participants, 37.0 years	Demographics, Lab data, COM		Cox regression, LR					MH/SH
Vehí et al. (95)	T1D	10 (41 years), 6 (40–60 years) participants	Insulin, carbohydrate intake, meals, physical activity, CGM parameters	CGM	GE, SVM, ANN	4 h for postprandial, 6 h for NH	Three consecutive SG < 70 or 54 mg/dL (15 min)	k-fold cross validation	Postprandial: Se = 69%, Sp = 80% (70 mg/dL): Se = 75.0%, Sp = 81.0% (54 mg/dL); NH: Se = 44.0%, Sp = 85.9%	Real-time, NH, Postprandial
Weiner et al. (96)	DM	6,745 participants, 55 years	Demographics, Lab data, GLA, COM, comorbidities, previous HYPO events, insurance		LR		Hypoglycemia < 70 mg/dL			MH/SH
Calhoun et al. (97)	T1D	127 participants	Demographics, Lab data, insulin, exercise intensity, daytime hypoglycemia	CGM	RF	Nighttime	Six consecutive SG ≤ 60 mg/dL (30 min)	5-fold cross validation	AUC = 0.622	NH
Ruan et al. (36)	DM	17,658 participants, 66 years	Demographics, medications, vital signs, Lab data, hospitalization procedure, previous HYPO events		XGBoost	Hospital stay	Hypoglycemia < 72 mg/dL, 54 mg/dL	10-fold cross validation	< 72 mg/dL: AUC = 0.96, Se = 70.0%, PPV = 88%; < 54 mg/dL: AUC = 0.96, Se = 67%, PPV = 97%	Inpatient
Elbaz et al. (98)	DM	3,605 (71 years), 6,060 (72.9 years) participants	Demographics, smoking, use of alcohol, comorbidities, Lab data, GLA, other medication		LR	First week of admission	Hypoglycemia ≤ 70 mg/dL	Internal, external	Validation set 1/2: AUC = 0.72/0.71	Inpatient
Wang et al. (99)	T1D	12 participants, 25.6 years	Insulin, carbohydrate absorption	CGM	Ruan model, Hovorka model	30 min	One single SG ≤ 70 mg/dL (15 min)	External	Validation: Acc = 95.97%, PPV = 91.77%, Se = 95.60%	Real-time, NH
Jermendy et al. (100)	DM	8,190 participants	Age, type of diabetes, GLA	SMBG	Description		Hypoglycemia ≤ 70 mg/dL			NH
Kyi et al. (101)	T2D	594 participants, 72 years	Demographics, GLA, hospital treatment factors, Lab data, comorbidities, observed-days		LR	Hospital stay	At least 2 days with capillary glucose < 72 mg/dL	Internal	AUC = 0.806, Se = 84.0%, Sp = 66.0%, PPV = 53.0%	Inpatient
Li et al. (102)	T1D, T2D	240 participants, 48.2 years		CGM	ARMA, RLS	30 min	Hypoglycemia ≤ 70 mg/dL	5-fold cross validation	Se = 95.72%	Real-time
Dave et al. (103)	DM	112 participants	Demographics, HbA1c, insulin, carbohydrate intake	CGM	LR, RF	30 min, 60 min	Hypoglycemia < 70 mg/dL	Hold-out validation	30 min: Se = 97.04%, Sp = 95.23%; 60 min: Se = 96.21%, Sp = 95.73%	Real-time
Yu et al. (104)	T2D	200 participants		CGM	Prefix Span	30 min	Hypoglycemia ≤ 54 mg/dL, ≤ 70 mg/dL, ≤ 79 mg/dL	Cross validation	≤ 54 mg/dL: Se = 85.9%; ≤ 70 mg/dL: Se = 80.36%; ≤ 79 mg/dL: Se = 78.07%	Real-time
Prendin et al. (105)	T1D	141 participants, > 18 years		CGM	AR, ARMA, ARIMA, SVR, RF, fNN, LSTM	30 min	One single SG < 70 mg/dL (5 min)	Random subsampling	Se = 82.0%, PPV = 64.0%	Real-time
Wenbo et al. (106)	DM	60 (44.8 years), 30 (24.4 years) participants		CGM	VMD, Kernel ELM, AdaBoost	60 min	Three consecutive SG < 70 mg/dL (15 min)	10-fold cross validation	Se = 94.8%, FPR = 7.7%	Real-time
Mathioudakis et al. (35)	DM	35,147 participants, 66 years	Demographics, diagnoses, insulin, hospitalization procedures, Lab data, medications, BG readings, heart rate		LR, RF, naïve Bayes, SGB	24 h after each glucose measurement	Hypoglycemia ≤ 70 mg/dL	Internal, external	Internal validation: c-statistic = 0.90; external validation: c-statistic: 0.86–0.88	Inpatient
Han et al. (107)	T2D	1,410 participants, 62.0 years	Demographics, medications, glycemic variability, Lab data	SMBG	LR	Perioperative period	Hypoglycemia < 70 mg/dL	Bootstrapping	AUC = 0.715	Inpatient
Witte et al. (108)	DM	38,250 participants, 64.3 years	Demographics, medications, Lab data		XGBoost	7 h	Hypoglycemia < 70 mg/dL	5-fold cross validation	Se = 59.0%. Sp = 98.8%, PPV = 71.8%	Inpatient
Yang et al. (109)	T2D	29,843 participants, 64.5 years	Demographics, medications, Lab data		XGBoost	Hospital stay	Hypoglycemia < 70 mg/dL	10-fold cross validation	AUC = 0.822, Acc = 0.93	Inpatient
Yun et al. (110)	T2D	2,645 participants, 62.8 years	Demographics, smoking, alcohol, physical activity, insulin, comorbidities, previous HYPO events, fasting glucose		ROC analysis	1 year	SH episodes requiring hospitalization or medical care	Internal, external	External validation: c-statistic = 0.878, Se = 83.3%, Sp = 84.7%	MH/SH
Wright et al. (111)	DM	6,279 participants, 57.0 years	Demographics, comorbidities, Lab data, vital signs, hospitalization orders, medications, glucose results		LR, RF, XGBoost	24 h	Hypoglycemia < 70 mg/dL within 24 h after insulin use	10-fold cross validation	LR: AUC = 0.81, Se = 44.0%; RF: AUC = 0.80, Se = 49.0%; XGBoost: AUC = 0.79, Se = 32.0%	Inpatient
Berikov et al. (112)	T1D	406 participants, 36.0 years	Demographics, previous HYPO events, IAH, insulin, CKD, COM, comorbidities, CGM-derived metrics	CGM	RF, LogRLasso, ANN	15 min, 30 min	Three consecutive SG < 70 mg/dL (15 min)	10-fold cross validation	15 min: AUC = 0.97, Se = 94.5%, Sp = 91.4%; 30 min: AUC = 0.942, Se = 90.4%, Sp = 87.4%	Inpatient, NH
Parcerisas et al. (113)	T1D	10 participants, 31.8 years	CGM raw data, meals, insulin, heart rate signal, steps, calories burned, sleep period	SMBG, CGM	SVM	Nighttime	Three consecutive SG < 70 mg/dL (15 min)	Leave-one-out validation, 5-fold cross validation	Population model: Se = 71%, Sp = 76% (include PA), Se = 70%, Sp = 72% (exclude PA); Individualized model: Se = 77.5%, Sp = 64.5% (include PA), Se = 73%, Sp = 75% (exclude PA)	NH
Wang et al. (114)	T2D	313 participants, 53.6 years	CGM-derived metrics, SMBG-derived metrics	SMBG	ROC analysis	Nighttime	Hypoglycemia < 70 mg/dL		AUC of predicting hypoglycemia using LAGE was 0.587, Se = 66.7%, Sp = 50%	NH
Tyler et al. (115)	T1D	20 participants, 34.5 years	CGM data, CGM-derived metrics, insulin, meal, heart rate, metabolic expenditure, age, height, weight	SMBG, CGM	MARS, LR, ARX	During aerobic exercise (4 h)	Hypoglycemia < 70 mg/dL	Hold-out validation, 20-fold cross validation	Population model: Se = 73%, Sp = 76%, Acc = 75% (Hold-out set); Se = 64%, Sp = 56%, Acc = 61% (20-fold CV); Personalized model: Se = 73%, Sp = 90%, Acc = 84% (Hold-out set); Se = 68%, Sp = 61%, Acc = 70% (20-fold CV)	During exercise
Duckworth et al. (116)	T1D	153 participants, 17.5 years	CGM data, CGM-derived metrics, age, sex, prior use of CGM, recent HbA1c	CGM	Heuristic model, LR, XGBoost	60 min	One single SG < 70 mg/dL (5 min)	5-fold cross validation	AUC = 0.998, average PPV = 95.3%	Real-time
Faccioli et al. (117)	T1D	11 participants	CGM data, insulin, meals	CGM	ARX	60 min	One single SG < 70 mg/dL (5 min)	Hold-out validation	PPV = 65%, Se = 88%	Real-time
Park et al. (118)	T1D	9 participants	CGM data, heart rate variability	CGM	SVM	10 min, 20 min, 30 min	Hypoglycemia < 70 mg/dL	Hold-out validation	Validation set: Se = 89.7%, Sp = 85.8%, Acc = 87.8% (10 min); Se = 88.0%, Sp = 84.3%, Acc = 86.2% (20 min); Se = 80.1%, Sp = 83.3%, Acc = 81.7% (30 min);	Real-time
Zhu et al. (119)	T1D	49 participants, >18 years	CGM data, carbohydrate, bolus insulin	CGM	FCNN, CRNN, LSTM, ARIMA, SVR, RF	30 min, 60 min	One single SG < 70 mg/dL (5 min)	Hold-out validation	FCNN model: Se = 84.09%, Sp = 65.60% (30 min); Se = 68.58%, Sp = 60.64% (60 min)	Real-time
Zhu et al. (120)	T1D	12 participants, 40.0 years	CGM data, non-invasive physiological data, carbohydrate, insulin	CGM	PKM, ARMA, SVR, ANN, LSTM, CRNN	60 min	Three consecutive SG < 70 mg/dL (15 min)	Leave-one-out validation	ARMA model: Acc = 88.58%, Se = 70.30%, Sp = 90.09%; PKM model: Acc = 87.20%, Se = 86.62%, Sp = 82.59%	Real-time

SMBG, self-monitoring blood glucose; CGM, continuous glucose monitoring; IFG, impaired fasting glucose; IGT, impaired glucose tolerance; T2D, type 2 diabetes; GLA, glucose-lowering agents; MH/SH, mild/severe hypoglycemia; T1D, type 1 diabetes; COM, diabetic complications; LR, logistic regression; HYPO, hypoglycemia; NR, not recorded; GFR, glomerular filtration rate; RF, random forest; SVM, support vector machine; k-nn, k-nearest neighbor; fNN, feed-forward neural network; ECG, electrocardiogram; ELM, extreme learning machine; NH, nocturnal hypoglycemia; ED, emergency department; DM, diabetes mellitus; ROC, receiver operating characteristics; CART, classification and regression trees; ANN, artificial neural networks; CKD, chronic kidney disease; PLS, partial least squares; DT, decision tree; ARIMA, autoregressive integrated moving-average; LSTM, long-short-term-memory; SVR, support vector regression; MLP, multilayer perceptron; LDA, linear discriminant analysis; MMTOP, multiple models for missing values at time of prediction; KRR, kernel ridge regression; GE, grammatical evolution; ANN, artificial neural networks; ARMA, autoregressive moving average; RLS, recursive least squares; VMD, variational mode decomposition; SGB, stochastic gradient boosting; AUC, area under the curve; Acc, accuracy; Se, sensitivity; Sp, specificity; PPV, positive predictive value; FPR, false positive rate; LogRLasso, logistic linear regression with Lasso regularization; MARS, multivariate adaptive regression spline; ARX, autoregressive model with exogenous inputs; FCNN, fast-adaptive and confident neural network; CRNN, convolutional recurrent neural network; PKM, physiologically-based kinetic model; SG, sensor glucose.

#### 3.1.1. Study participants

**Figure 3:**
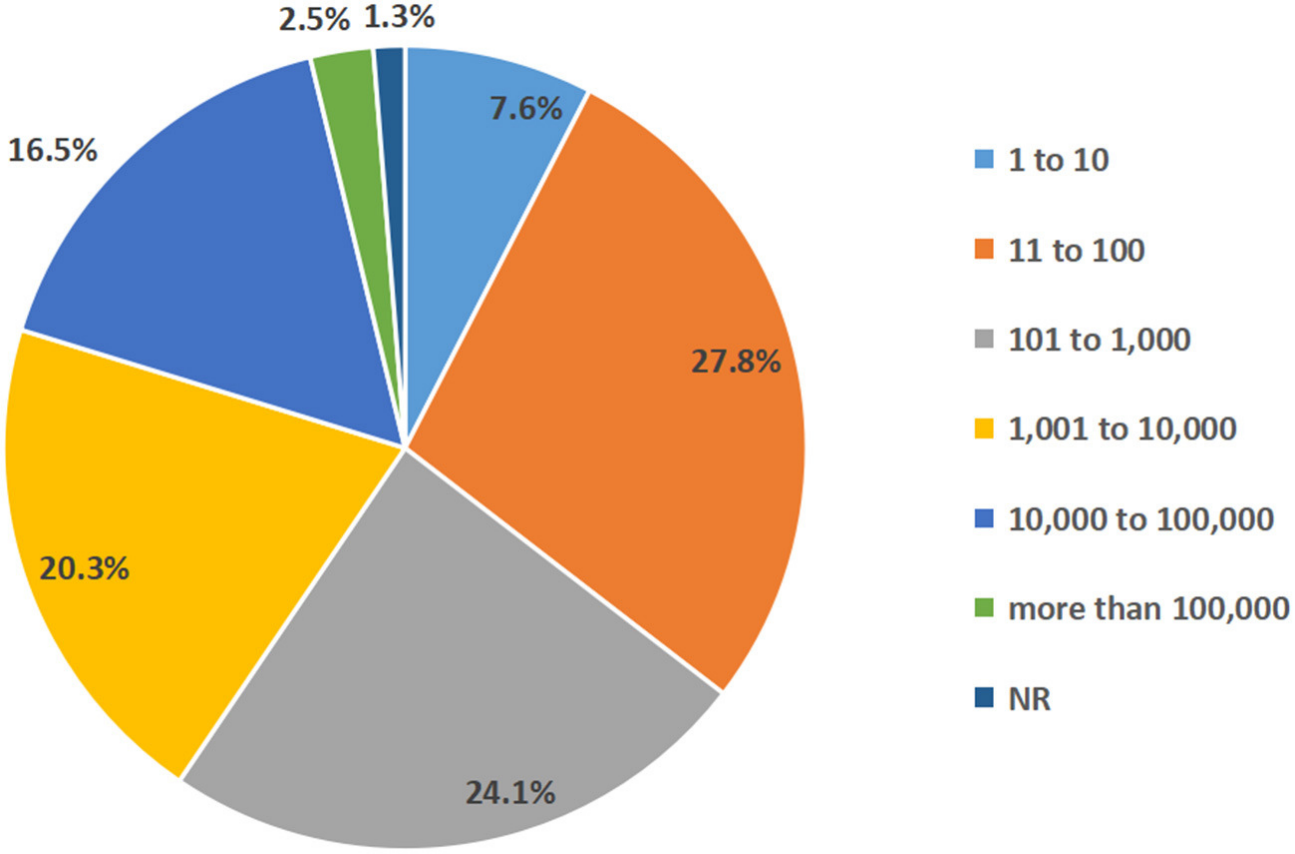
The sample size of participants and proportion of selected studies.

Patients with T1D and T2D made up the majority of the participants in the chosen studies, with 31 of them (39.2%) only including T1D patients. The sample sizes of among studies varied significantly: Six studies with a maximum of 10 participants (7.6%), 22 studies with 11 to 100 participants (27.8%), 19 studies with 101 to 1,000 participants (24.1%), 16 studies with 1,001–10,000 participants (20.3%), 13 studies with 10,001–100,000 participants (16.5%) and two studies more than 100,000 participants (2.5%) (Figure 3). The study subjects were from all age groups.

#### 3.1.2. Inputs

**Figure 4:**
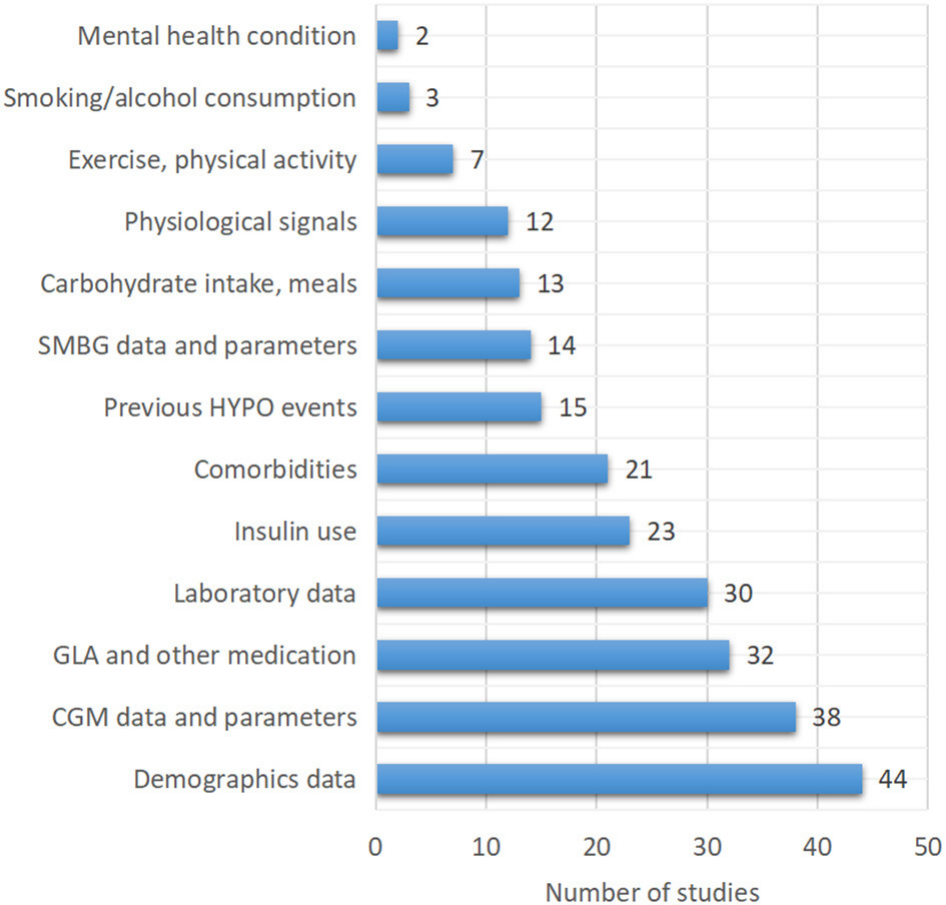
The type and number of input parameters used to train the models.

One of the most important strategies for successful hypoglycemia prediction is the selection of appropriate inputs. Thirty-eight of the 79 included studies (48.1%) used CGM data or CGM-derived indices to predict hypoglycemia. Demographics information, insulin use, laboratory tests results, comorbidities, and a history of hypoglycemia were all widely included in clinical hypoglycemia prediction models. Furthermore, other blood glucose-related factors such as carbohydrate intake or meal information (13/79, 16.5%) and physical activity or exercise (7/79, 8.9%) were also included. In addition to the aforementioned factors, 12 studies (15.2%) used physiological parameters such as heart rate, near-infrared light, skin impedance, skin temperature, sweating, and sleep to develop hypoglycemia prediction models (Table 2, Figure 4).

**Table 2:** Clinical and glucose inputs for model training.

**Input type**	**References**
Demographics data	(35, 36, 44–46, 48, 50, 57–67, 71, 72, 74, 77, 81, 83–85, 89, 91, 92, 94, 96–98, 100, 101, 103, 107–112, 115, 116)
CGM data and parameters	(43, 47, 49, 54–56, 61, 63, 68, 70, 73, 76, 78–80, 82, 83, 85, 88, 90, 93, 95, 97, 99, 102–106, 112–120)
GLA and other medication	(35, 36, 44–46, 48, 50–52, 58–60, 62–64, 66, 67, 71, 74, 81, 83, 89, 91, 92, 96, 98, 100, 101, 107–109, 111)
Laboratory data	(35, 36, 44–46, 51, 58–61, 63, 66, 67, 71, 74, 81, 83, 84, 89, 91, 92, 94, 96–98, 101, 107–109, 111)
Insulin use	(35, 57, 64, 67, 71–73, 82, 85, 92, 93, 95–97, 99, 103, 110, 112, 113, 115, 117, 119, 120)
Comorbidities	(44–46, 58–60, 62, 64, 66, 67, 71, 84, 87, 91, 92, 96, 98, 101, 110–112)
Previous HYPO events	(36, 46, 48, 58, 59, 62, 64, 71, 74, 81, 91, 92, 96, 110, 112)
Carbohydrate intake, meals	(72, 73, 82, 85, 93, 95, 99, 103, 113, 115, 117, 119, 120)
SMBG data and parameters	(35, 46, 52, 53, 61, 66, 67, 69, 82, 100, 107, 113–115)
Physiological signals	(35, 47, 53, 65, 68, 69, 75, 82, 113, 115, 118, 120)
Exercise, physical activity	(64, 70, 77, 83, 95, 97, 110)
Smoking/alcohol consumption	(64, 98, 110)
Mental health condition	(45, 89)

CGM, continuous glucose monitoring; GLA, glucose-lowering agents; HYPO, hypoglycemia; SMBG, self-monitoring of blood glucose.

#### 3.1.3. Prediction horizon (PH)

PH is the time period that the model has to forecast the outcome in the future. Prediction windows ranging from 15 min to hours, days, or even months have been reported. It is natural to anticipate a decrease in prediction power as the PH increases due to the limited number of available confounding factors in the data used to train the model. A shorter PH may be more useful for prompt clinician intervention, whereas a larger PH increases the prevalence of an outcome and consequently model performance, but may be less useful as a decision support tool (42). An increase in PH, on the other hand, improves clinical usability of prediction services by extending the time required to take the necessary action during a critical situation, but at the expense of clinical accuracy. According to the aforementioned PH division, 21 studies were short-term forecasting (26.6%), 21 studies were mid-term forecasting (26.6%) and 19 studies were long-term forecasting (24.1%).

#### 3.1.4. Modeling approaches

**Figure 5:**
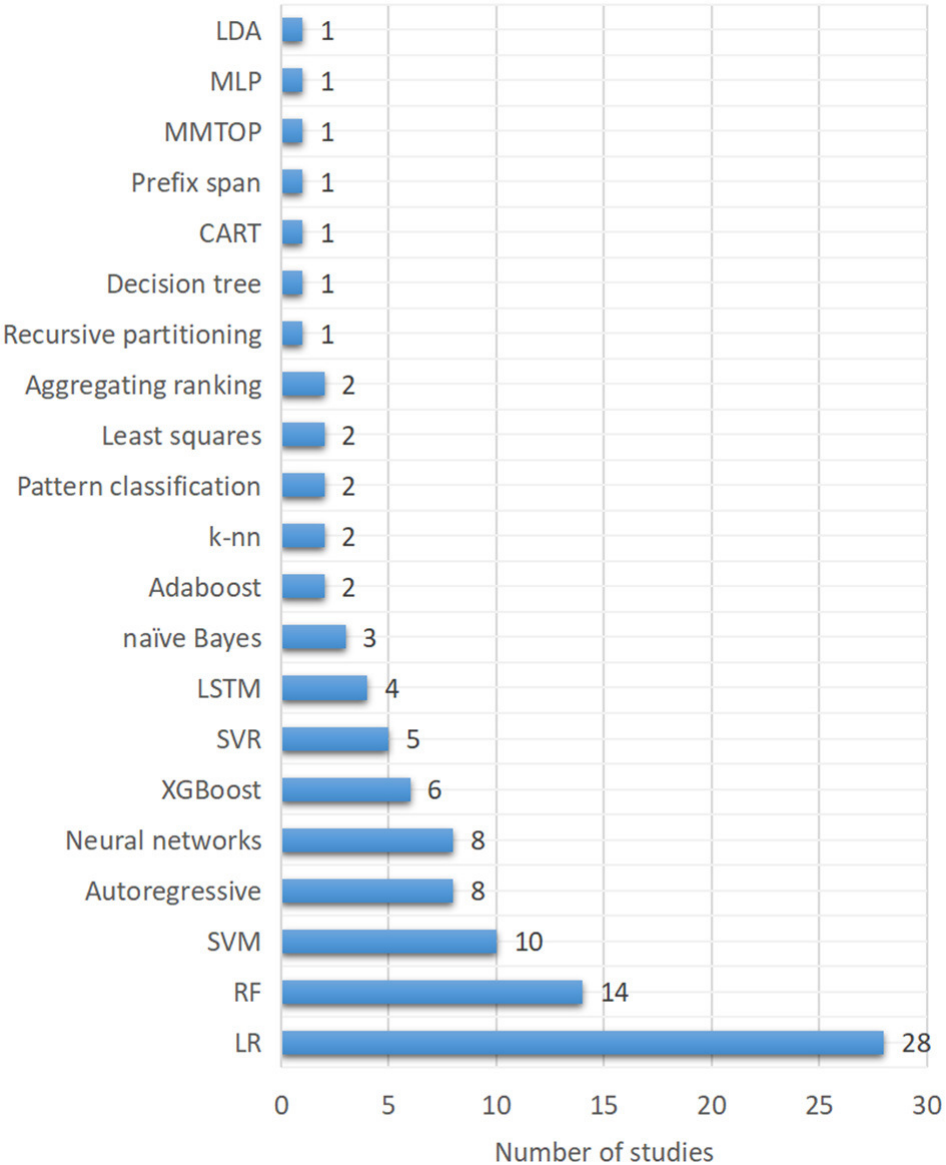
Classes of machine learning techniques used in the modeling of hypoglycemia prediction.

Various classes of ML techniques have been used in general dynamic system modeling, regression, and prediction services. However, for hypoglycemia prediction in the present study, logistic regression (LR) were the most used techniques (28/79, 35.4%), as shown in Figure 5. Random forest (RF), one of the tree-based models, was the second most used approach (14/79, 17.7%). Support vector machines (SVM) was the third most used algorithm (10/79, 12.7%). Autoregressive and neural networks in various forms ranked as the fourth most used techniques (8/79, 10.1%). XGBoost and support vector regression (SVR) ranked as the fifth (6/79, 7.6%) and sixth most used technique (5/79, 6.3%). Long-short-term memory (LSTM) (4/79, 5.1%), naïve Bayes (3/79, 3.8%), Adaboost (2/79, 2.5%), k-nearest neighbors (k-nn) (2.5%) and other techniques were also used in hypoglycemia prediction.

#### 3.1.5. Model validation

Model validation is crucial for algorithm development and performance estimation. External validation is important since internal validation may overestimate model performance on a future cohort of patients (121). There are 12 studies utilizing external validation in the present study (15.2%). Internal validation strategies include re-substitution validation, hold-out validation, k-fold cross-validation, leave-one-out cross-validation, and repeated k-fold cross-validation (122). The most commonly used strategies in the reviewed articles are various forms of k-fold cross-validation (22/79, 27.8%). The K-fold cross-validation strategy involves randomly partitioning the datasets into k equal subsets and using one set as a validation set and the rest for training, repeating the process for each subset. Moreover, random subsampling, bootstrapping, hold-out validation, leave-one-out validation approaches are also used.

#### 3.1.6. Performance metrics

As model evaluation performance measures, the majority of studies used sensitivity, specificity, and area under the curve (AUC). For prediction models based on clinical data, correlation coefficients and c-statistics were also frequently used. In addition, accuracy, positive predictive value (PPV) and false positive rate (FPR) were used as evaluation parameters. Each metric was defined in Supplementary Table S1.

### 3.2. Real-time hypoglycemia prediction

The CGM system can detect the glucose concentration in the interstitial fluid continuously and comprehensively, laying the groundwork for modern glucose monitoring and the emergence of AP. A CGM system, an insulin pump, and a dosing algorithm comprise AP (123). Algorithms are used to implement individual-based accurate blood glucose prediction. The proposal and improvement of dosing algorithms have gradually evolved into a bottleneck of AP development with the increasing maturity of CGM systems. Accurate prediction of impending hypoglycemia is difficult due to large intra- and inter-subject variability, as well as numerous exogenous factors such as diet, exercise, hypoglycemic drugs, and mood changes that can affect blood glucose levels (124). Following a review of the literature, it was discovered that the current impending hypoglycemia prediction primarily includes physiological models, data-based models, and hybrid models (43). Simply put, the development of physiological models is dependent on an understanding of glucose metabolism in the body. These models, which are often compartment models, simulate glucose metabolism and can be used to study glucose-regulated physiological processes. Data-driven models, on the other hand, rely primarily on CGM data and occasionally on additional signals to simulate the patient's physiological response without involving physiological variables. Hybrid models typically combine a physiological model with a data-driven model. Such models incorporate dietary information and insulin absorption *via* physiological models, as well as massive CGM data, to improve the overall prediction accuracy of this model.

#### 3.2.1. Only CGM data as inputs for real-time hypoglycemia prediction

This section presents the most recent research on data-based and hybrid hypoglycemia prediction models that only use CGM data as inputs (78, 79, 90, 102, 104–106). AR models were commonly used. Yang et al. (78) proposed an AR integrated moving average (ARIMA) model with an adaptive recognition algorithm. After training with CGM data from 100 subjects (50 T1D + 50 T2D), it was discovered that this model had 100.0% sensitivity in predicting hypoglycemic events, a 9.4% FPR, and an early alert with an average 25.5 min treatment time window to avoid hypoglycemia deterioration. Gadaleta et al. (79) used CGM data from 89 T1D patients to compare current common ML models (static and dynamic) and discovered that the SVR model performed best in terms of prediction accuracy as well as the speed of hypoglycemia detection, with sensitivity and PPV of 75.0 and 51.0%, respectively. Another study comparing linear and nonlinear models laterally found that at a PH of 30 min, the individualized ARIMA model outperformed all linear models in terms of hypoglycemic event detection and prediction accuracy (105). Furthermore, Marcus et al. (90) used a novel patient-specific supervised machine learning (SML) model for hypoglycemia prediction after 30 min and discovered that when the best-fit model was selected for each patient, the hypoglycemia sensitivity was 64.0%, and FPR was 4.0%. Even when only CGM glucose data below 70 mg/dL were included, similar results were obtained. Li (102) and Yu et al. (104) used the model of change detection method and the Winsorization method in conjunction with the autoregressive moving average (ARMA) model and the recursive least squares (RLS) method, respectively. The sensitivity of the former was 95.72%, and the sensitivities of the latter were 85.90, 80.36, and 78.07% when the threshold of hypoglycemia was set at 54, 70, and 79 mg/dL, respectively. Wenbo et al. (106) achieved 94.80% sensitivity for hypoglycemic event prediction at a PH of 60 min using the variational mode decomposition (VMD)-kernel extreme learning machine (KELM)-AdaBoost algorithm. As for sensitivity, the authors of (78) achieved the highest sensitivity at a PH of 30 min (Table 3).

**Table 3:** Machine learning approaches for real-time hypoglycemia prediction and best results performed.

**References**	**Participants, type**	**Inputs**	**PH (min)**	**Algorithm**	**Threshold**	**Validation**	**Performance**
Gadaleta et al. (79)	89, T1D	CGM	30	SVR	≤ 70 mg/dL	Leave-one-out validation	Se = 75.0%, PPV = 51.0%
Li et al. (102)	240, T1D + T2D	CGM	30	ARMA, RLS	≤ 70 mg/dL	5-fold cross validation	Se = 95.72%
Marcus et al. (90)	11, T1D	CGM	30	KRR	< 70 mg/dL	Hold-out validation	Se = 64.0%, FPR = 4.0%
Prendin et al. (105)	141, T1D	CGM	30	Individualized ARIMA	< 70 mg/dL	Random subsampling	Se = 82.0%, PPV = 64.0%
Yang et al. (78)	100, T1D + T2D	CGM	30	ARIMA	≤ 70 mg/dL		Se _T1D/T2D_ = 100.0/100.0%; FPR _T1D/T2D_ = 10.7/8.0%
Yu et al. (104)	200, T2D	CGM	30	Prefix Span	≤ 54 mg/dL, ≤ 70 mg/dL, ≤ 79 mg/dL	Cross validation	≤ 54 mg/dL: Se = 85.9%; ≤ 70 mg/dL: Se = 80.36%; ≤ 79 mg/dL: Se = 78.07%
Wenbo et al. (106)	60, DM	CGM	60	VMD-KELM-AdaBoost	< 70 mg/dL	External	Se = 94.8%, FPR = 7.7%
Cichosz et al. (47)	21, T1D	CGM, HRV	20	Pattern classification	< 70 mg/dL	10-fold cross validation	AUC = 0.96, Se = 100%, Sp = 91%
Cichosz et al. (68)	56, T1D	CGM, HRV	20	Pattern classification	≤ 70 mg/dL	Internal	AUC = 0.95
Park et al. (118)	9, T1D	CGM, HRV	30	SVM	< 70 mg/dL	Hold-out validation	Se = 80.1%, Sp = 83.3%, Acc = 81.7%
Dave et al. (103)	112, DM	CGM, INS, CHO	30, 60	LR, RF	< 70 mg/dL	Hold-out validation	Se = 97.04%, Sp = 95.23% (30 min); Se = 96.21%, Sp = 95.73% (60 min)
Faccioli et al. (117)	11, T1D	CGM, INS, CHO	60	ARX	< 70 mg/dL	Hold-out validation	PPV = 65%, Se = 88%
Zhu et al. (119)	49, T1D	CGM, INS, CHO	30, 60	FCNN	< 70 mg/dL	Hold-out validation	Se = 84.09%, Sp = 65.60% (30 min); Se = 68.58%, Sp = 60.64% (60 min)
Zhu et al. (120)	12, T1D	CGM, INS, CHO	60	PKM	< 70 mg/dL	Leave-one-out validation	Acc = 87.20%, Se = 86.62%, Sp = 82.59%
Duckworth et al. (116)	153, T1D	CGM, age, sex, HbA1c	60	XGBoost	< 70 mg/dL	5-fold cross validation	AUC = 0.998, average PPV = 95.3%

T1D, type 1 diabetes; T2D, type 2 diabetes; DM, diabetes; CGM, continuous glucose monitoring; HRV, heart rate variability; CHO, carbohydrate intake; INS, insulin.

#### 3.2.2. Combining CGM data, insulin, dietary intake and physical activity as inputs

In addition to using CGM data and insulin to predict, carbohydrate intake was also used in models (Table 3). Dave et al. (103) confirmed the benefits of including blood glucose-affecting physiological factors in the model by demonstrating satisfactory sensitivity and specificity in hypoglycemia prediction. A recent study indicated a promising predictive efficacy of physiologically-based kinetic model (PKM) which integrated CGM data, carbohydrate intake and insulin usage (120). Additionally, one of the significant factors affecting blood glucose is physical activity (exercise) (125). However, the effect of physical activity (exercise) on blood glucose varies considerably based on many factors, such as the type of activity, amount and intensity of activity, and duration. When compared to studies that only used CGM data as inputs, the contribution of features (meal, insulin, and exercise) other than CGM glucose data is lower but not insignificant; their significance rises for predictions with PH of 60 min (103). Hence, adding clinical factors other than CGM data may allow for improved predictive efficacy, even though overall model performance had not improved to a satisfactory degree.

#### 3.2.3. Non-invasive sensors in hypoglycemia prediction

Aside from CGM data, some researchers have also used non-invasive methods to predict hypoglycemia. Cichosz et al. (47, 68) used CGM and electrocardiograph (ECG) data to predict short-term hypoglycemia events, achieving an AUC of more than 0.96 and a sensitivity of 100% when the PH was set at 20 min. However, the sensitivity and specificity of another NN model for predicting hypoglycemia based solely on ECG signals were only 78.0 and 60.0%, respectively, which could be explained in part by the limited glucose data used in modeling (53). Furthermore, Elvebakk et al. (69) collected physiological parameters such as ECG signals, near-infrared light (NIR), and skin impedance from 20 T1D patients with IAH undergoing hypoglycemic clamp, and they demonstrated that a probability model based on the physiological signals described above could identify 88% of hypoglycemia. Similarly, Tronstad et al. (75) compared the accuracy of partial least squares (PLS) and artificial NN (ANN) in predicting hypoglycemia using NIR signals, skin impedance, and skin temperature data from the same patient source. Their findings showed that the ANN model that combined NIR, skin temperature, and skin impedance outperformed. However, in general, NIR and bioimpedance-based hypoglycemia detection was not very accurate, but it could provide blood glucose trends to some extent.

In summary, current studies aimed at predicting real-time hypoglycemia were mostly short-term. As the PH increased, the prediction accuracy inevitably decreased. Hence, accurate long-term hypoglycemia prediction is extremely urgent. Furthermore, hypoglycemia prediction is gradually shifting from a single signal to a combination of signals to more accurately simulate real-life glucose changes. As shown in Table 3, there was no single technique that could be identified as the most popular model in terms of algorithms. The ML approaches trend revealed that researchers were still experimenting with a diverse set of ML techniques. It can be concluded that there have been some promising results in the field of real-time hypoglycemia prediction, but there is still much room for advancement.

### 3.3. Mild and severe hypoglycemia prediction

#### 3.3.1. Clinical predictors of hypoglycemia and prediction models based on clinical parameters

Prior to the booming of ML in hypoglycemia prediction, simple predictions based on massive clinical data emerged in this field of study (Table 4). Long duration of diabetes, alcohol consumption, eating disorders, low BMI, insulin use, low LDL levels, combined diabetic retinopathy (DR) or diabetic peripheral neuropathy (DPN), depression or dementia, great glycemic variability, infection, and heart failure were identified as risk factors for overall hypoglycemic events (44–46, 49, 51, 63, 96). A long-term SH risk prediction study based on clinical parameters in 1,676, 885 adult T2D patients found that old age, female gender, smoking, alcohol abuse, low BMI, lack of exercise, history of SH, use of insulin or multiple oral hypoglycemic agents, combined hypertension and chronic kidney disease (CKD), long duration of diabetes, and a high Charlson comorbidity index were all important risk factors for the development of SH (64). Several studies have shown an association between insulin use, intensive insulin therapy, previous history of SH, and SH (44, 48, 51, 62, 91). Furthermore, black race, sulfonylurea use, low HbA1c, low serum creatinine levels, and poor cognitive function were also identified as risk factors for SH (44, 91). Hu et al. (84) performed ROC analysis on fasting insulin, fasting blood glucose, and total insulin treatment time of 257 T2D patients receiving intensive therapy and reported an AUC of 0.666 in hypoglycemia prediction. Karter et al. (57) classified 165,148 patients into high, medium, and low-risk groups for SH hospitalization by assessing six factors: number of previous hypoglycemia hospitalizations, insulin use, sulfonylurea use, previous emergency history, CKD stage, and age. Following model validation, they concluded that the proposed SH risk assessment tool was accurate and effective after 12 months of observation. Accordingly, some researchers have used another 6-parameter model (age, type of diabetes, HbA1c, eGFR and previous history of hypoglycemia) to predict the 6-month SH risk (58).

**Table 4:** Clinical predictors of mild/severe hypoglycemia.

**Category of predictors**	**Predictors of hypoglycemia**	**Predictors of SH**
Demographics	• BMI < 30 kg/m^2^ (46), lower BMI (44) • Younger age (44) • Drinking (51, 96) • Longer diabetes duration (63) • Black race (96) • Eating disorder (96)	• Black race (91, 92) • Older age (44, 64, 110) • Female (64) • Current smoker (64) • Drinking (64, 92) • Longer diabetes duration (64, 110) • lower BMI (64, 110)
GLA	• Insulin use > 10 years (46), insulin (96) • 2 injections/day (46) • SU use with antibiotics (96)	• Insulin use (62, 64, 91), intensive glucose control (51, 62) • Multiple OHA use (64) • SU use (91)
CGM parameters	• Higher SD, MAG, MAGE, CV (49, 63), low MBG, higher LBGI (63)	
Other	• Diabetic retinopathy (45), diabetic neuropathy (96) • Low LDL-c level (45) • Altered mini-Geriatric Depression Scale (45) • Previous hypoglycemia (46, 96) • Infection within 30 days (96) • Chronic heart failure (96) • Dementia or falls (96) • HbA1c ≤ 6.5% (96)	• Previous SH events (48, 64, 91, 92) • Lack of exercise (64) • Presence of hypertension (44, 64), antihypertensive medication use (62) • CKD (64, 92) • High Charlson score (64) • HbA1c < 7% (91) • High serum creatinine level (44) • Low cognitive function (44) • Depression or other psychiatric disorders (92) • Medicaid insurance (92) • History of CVD (92) • Lower eGFR (110) • Higher albuminuria (110)

SH, severe hypoglycemia; GLA, glucose-lowering agents; OHA, oral hypoglycemic agents; SU, sulfonylurea; CGM, continuous glucose monitoring; SD, standard deviation of glucose; MAG, mean absolute glucose; MAGE, mean amplitude of glucose excursions; CV, coefficient of variance; MBG, mean blood glucose; LBGI, low blood glucose index; CKD, chronic kidney disease; CVD, cardiovascular disease.

A remarkable cohort study of 27,841 T1D patients followed for an average of 7 years to model T1D health outcomes revealed that male gender, Ln (HbA1c), HDL level, and smoking were risk factors for hypoglycemia (HR = 1.32, 1.63, 1.14, and 1.40, respectively), while Ln (eGFR) was a protective factor (HR = 0.77) (94). Another SH predictive model study based on electronic health record data of 47, 280 T2D patients found that a history of previous hypoglycemia (HR = 4.44), black race (HR = 1.81), Medicaid insurance (HR = 1.35), previous history of cardiovascular disease (HR = 2.35), depression (HR = 1.28), mental disorder other than depression (HR = 1.55), alcohol consumption (HR = 1.55), and CKD (HR = 1.86) were risk factors for SH, while the relationship between sulfonylureas and SH changed with HbA1c: sulfonylurea use was a risk factor (HR = 1.61) when HbA1c was 6%, but it became a protective factor (HR = 0.69) when HbA1c was 9%. Furthermore, the effect of HbA1c levels on SH varied at some extreme values: when the reference HbA1c was 6%, the corresponding HR was 1.59 when an HbA1c level of 5%, however, HR of HbA1c changed to 0.73 when at a level of 7%, and the HR for HbA1c = 9% was 1.39 instead (92).

#### 3.3.2. Application of machine learning to predict hypoglycemia

In addition to clinical models, there were a number of studies that used ML approaches to predict mild and severe hypoglycemia. Ma et al. (89), for example, proposed a model to predict SH that could handle missing data, and they finally included 48 risk factors associated with SH such as demographic data (age, ethnicity, education information), vital signs (diastolic blood pressure, DBP), laboratory test results (creatinine, eGFR, urine protein, UACR) and medication regimens (diuretics, potassium supplements, ACEI, α-blockers, β-blockers, anticoagulants, sulfonylureas, biguanides, thiazolidinediones, insulin, etc.) after univariate analysis for relevant variable screening and showed that the average c-statistic of their proposed SH prediction model was 0.77. Predictive modeling also made use of mathematical approaches. Samuel et al. (50) identified four clinical factors associated with MH (BMI, diabetes duration, HbA1c, and GFR) and proposed a mathematical formula for calculating the incidence of MH incorporating these four parameters, but its accuracy was highly uncertain in different populations of patients with diabetes. However, the prediction efficiency of ML was not always superior to that of traditional statistical methods. Li et al. (71) used electronic health records data from 38,780 patients with diabetes to create a prediction model for hypoglycemia and discovered that the ML method (RF) was only 1% more effective than LR in predicting hypoglycemia. A robust recursive partitioning algorithm based SH-related emergency department (ED) use prediction model was proposed based on a large sample size and external validation (57) (Table 5).

**Table 5:** Machine learning approaches for MH/SH prediction and best results performed.

**References**	**Participants, type**	**Inputs**	**PH**	**Algorithm**	**Outcome**	**Validation**	**Performance**
Sudharsan et al. (52)	163, T2D	SMBG, medication	24 h, the 8th day	RF, SVM	Hypoglycemia < 70 mg/dL	Cross validation	24 h: Se = 91.7%, Sp = 69.5%;8th day: Se = 90.4%, Sp = 91.1%
Karter et al. (57)	206,435, T2D	Demographics, insulin/SU use, history of HYPO-related utilization, prior year ED use	12 months	Recursive partitioning	SH-related ED or hospital use	Internal, external	IV: c-statistic = 0.83, EV 1/2: c-statistic = 0.81/0.79
Li et al. (71)	38,780, DM	Demographics, Lab data, previous HYPO events, GLA, comorbidities, COM, insurance	2 years	RF	Hypoglycemia < 70 mg/dL	10-fold cross validation	AUC = 0.90
Elhadd et al. (83)	13, T2D	CGM, demographics, Lab data, PA, medication	During Ramadan	XGBoost			Acc = 27.9%
Ma et al. (89)	10,251, T2D	Demographics, Lab data, medications, physical exam findings, mental health results		MMTOP	Hypoglycemia < 50 mg/dL	10-fold cross validation	C-statistic = 0.77
Misra-Hebert et al. (91)	1,876, T2D	Demographics, Lab data, comorbidities, GLA, previous HYPO events	3 months	LR	SH events identified by diagnosis code	Bootstrapping	AUC = 0.89, Se = 82.0%, Sp = 79.0%

T2D, type 2 diabetes; DM, diabetes; SMBG, self-monitoring of blood glucose; CGM, continuous glucose monitoring; HYPO, hypoglycemia; Lab, laboratory; GLA, glucose-lowering agents; PA, physical activity; IV, internal validation; EV, external validation.

### 3.4. Nocturnal hypoglycemia (NH) prediction

#### 3.4.1. Prediction of NH based on clinical parameters

Age between 10.0 and 19.9 years, diagnosis of T1D, and initiation of insulin therapy were found to be risk factors for NH events (< 54 mg/dL, 00:00–05:59) in a study using self-monitoring of blood glucose (SMBG) values and clinical indicators from 8,190 patients with diabetes (100). For NH prediction, CGM parameters had also been adopted in addition to clinical parameters. Daytime (6:00-22:59) mean absolute glucose (MAG) and mean pre-midnight blood glucose levels had predictive value for NH events, according to a cross-sectional study of 83 insulin-treated hospitalized T2D patients (56). Sakurai et al. (61) developed an equation relating age, SMBG values, and basal insulin dose to predict NH. Besides, strenuous physical activity was found to be an important predictor of NH after adjusting for age and gender (70) (Table 6).

**Table 6:** Clinical predictors of nocturnal hypoglycemia.

**Category of predictors**	**Predictors**
Demographics	• Age (10.0–19.9 years) (100)
	• Type 1 diabetes (100)
GLA	• Insulin treatment (100)
CGM parameters	• Daytime Mean Absolute Glucose (MAG) (56)
	• Pre-midnight mean glucose (56)
Other	• Vigorous intensity physical activity (70)

GLA, glucose-lowering agents; CGM, continuous glucose monitoring.

#### 3.4.2. Application of ML to predict NH

ML approaches based on CGM data were widely used for NH prediction in addition to clinical factors (Table 7). Tkachenko (55) and Sampath et al. (54) proposed combining predictive risk factors of NH based on CGM data, which both resulted in improved predictive performance. Based on massive nocturnal CGM raw glucose data derived from 9,800 T1D patients, a random forest (RF) model demonstrated an overall NH predictive performance of AUC up to 0.84 (AUC = 0.90 during 00:00–03:00, and AUC = 0.75 during 03:00–06:00) (76). Furthermore, a novel CGM metric-gradient and combining mean sensor glucose enabled the prediction of NH events in patients with diabetes with a sensitivity of 92.05% and a false positive rate of 7.69% (88).

**Table 7:** Machine learning approaches for NH prediction and best results performed.

**References**	**Participants, type**	**Inputs**	**PH**	**Algorithm**	**Threshold**	**Validation**	**Performance**
Sampath et al. (54)	34, T1D	CGM raw data and parameters	Nighttime	Aggregating ranking	< 70 mg/dL	External	Se = 77.0%, Sp = 83.4%
Tkachenko et al. (55)	34, T1D	CGM raw data and parameters	Nighttime	Aggregating ranking	< 70 mg/dL	Random subsampling	Se = 73.4%, Sp = 87.8%
Klimontov et al. (56)	83, T2D	CGM raw data and parameters	Nighttime	LR	≤ 70 mg/dL		Acc = 75.6%, Se = 84.0%, Sp = 62.1%
Vu et al. (76)	9,800 T1D	CGM	3 h, 6 h	RF	< 70 mg/dL	10-fold cross validation	3h: AUC = 0.90; 6h: AUC = 0.84
Jensen et al. (85)	463, T1D	CGM, demographics, INS, CHO	Nighttime	LDA	≤ 54 mg/dL	5-fold cross validation	AUC = 0.79, Se = 75.0%, Sp = 70.0%
Mosquera-Lopez et al. (93)	134, T1D	CGM, INS, CHO	Nighttime	SVR	< 70 mg/dL	External	AUC = 0.86, Se = 94.1%, Sp = 72.0%
Calhoun et al. (97)	127, T1D	CGM, demographics, Lab data, INS, PA, daytime HYPO	Nighttime	RF	≤ 60 mg/dL	5-fold cross validation	AUC = 0.622
Parcerisas et al. (113)	10, T1D	CGM raw data, PA, CHO, INS, heart rate signal, steps, calories burned, sleep period	Nighttime	SVM	< 70 mg/dL	Leave-one-out, 5-fold cross validation	Population model: Se/Sp = 71/76% (include PA) Individualized model: Se/Sp = 73/75% (exclude PA)
Bertachi et al. (82)	10, T1D	CGM raw data, CHO, INS, heart rate signal, steps, calories burned, sleep period	Nighttime	SVM	< 70 mg/dL	5-fold cross validation	Acc = 80.77%, Se = 78.75%, Sp = 82.15%
Li et al. (88)	1,921, T1D + T2D	CGM	Nighttime	LSTM	≤ 70 mg/dL	Internal	Se = 92.05%, FPR = 7.69%
Vehí et al. (95)	16, T1D	CGM, INS, CHO, PA	6 h	ANN	< 70 mg/dL	k-fold cross validation	Se = 44.0%, Sp = 85.9%
Wang et al. (99)	12, T1D	CGM, INS, CHO	30 min	GIM	≤ 70 mg/dL	External	Validation: Acc = 95.97%, PPV = 91.77%, Se = 95.60%
Berikov et al. (112)	406, T1D	CGM, demographics, previous HYPO, IAH, INS, CKD, COM, comorbidities	15 min,; 30 min	RF	< 70 mg/dL	10-fold cross validation	15 min: AUC = 0.97, Se = 94.5%, Sp = 91.4%; 30 min: AUC = 0.942, Se = 90.4%, Sp = 87.4%

T1D, type 1 diabetes; T2D, type 2 diabetes; CGM, continuous glucose monitoring; INS, insulin; CHO, carbohydrate intake; PA, physical activity; HYPO, hypoglycemia; IAH, impaired awareness of hypoglycemia; CKD, chronic kidney disease; COM, diabetic complications; Lab, laboratory; GIM, glucose-insulin mixture model.

Models that included blood glucose-affecting physiological factors other than CGM data were also widely used in the field of NH prediction. Jensen et al. (85), for example, performed ML feature extraction and ROC curve analysis on basic demographic data, dietary intake, and insulin use combined with CGM data from 463 T1D patients and discovered that the combined extracted CGM indices (linear regression slope of blood glucose during 21:00–24:00, lowest blood glucose value, lowest blood glucose value on the previous night) and BMI could achieve an AUC of 0.79 for NH prediction (sensitivity: 75%, specificity: 70%). Furthermore, Bertachi et al. (82) confirmed the importance of combining physical activity with CGM data to predict NH events: the specificity of predicting NH was significantly improved to 91.9% when additional information such as heart rate, number of steps, calorie expenditure, and sleep were added. Calhoun et al. (97) discovered an association between NH and bedtime BG, exercise intensity, daytime hypoglycemia, HbA1c, and active insulin (insulin on board, IOB). Vehi et al. (95) established an ANN model that included CGM data, exercise and sleep information from six T1D patients and had a 44.0% sensitivity and an 85.9% specificity in predicting NH. Another study of NH prediction using a SVR model based on CGM data found that blood glucose values at bedtime, age, and mean blood glucose 1 h before bedtime were related to the occurrence of NH, and the AUC of this model to finally predict NH events was 0.86 (sensitivity: 94.1%, specificity: 72.0%) (93).

It can be seen that the most popular approaches for NH prediction in our reviewed articles were RF and SVM. The RF model including CGM, demographics data, INS and other clinical indicators of 406 T1D patients established by Berikov et al. (112) achieved a better performance at a PH of 30 min. Whereas a SVR model taking CGM, insulin use and carbohydrate intake information into consideration showed satisfactory AUC (0.86) and sensitivity (94.1%) for a longer prediction window (93).

Seventy-five percent of hypoglycemic events associated with coma or seizures occur at night, as warning autonomic symptoms caused by hypoglycemia are frequently insufficient to awaken the patient (126). Since NH events are urgent and harmful to patients, current NH prevention focuses primarily on accurately predicting upcoming NH events and alerting using modern glucose monitoring technology and AP to urge medical staff or patients to take prompt action. The findings of our literature review also highlight recent significant advances in the field of NH prediction using ML, however, these algorithms and models must still be validated in a large sample and tested in real-world applications in the future.

### 3.5. Inpatient hypoglycemia prediction

A recent electronic health record-based risk prediction study for iatrogenic hypoglycemic events included 35,147 hospitalized patients (mean age, 66 years) who received at least 1 U of insulin and completed four finger stick records (35). Demographic data, diagnostic information, inpatient procedure, laboratory test results, finger blood results, and therapeutic drugs were among the 43 types of data extracted from electronic health records. They revealed that basal insulin dose, CV of finger stick blood values, previous hypoglycemic events, nadir glucose value, body weight, and mean blood glucose in the first 24 h of hospitalization were the most important predictors of hypoglycemia. The c-statistic was 0.90 of internal validation and was 0.86–0.88 of external validation (35). Stuart et al. (59) used multivariate LR to develop a prediction model from hospital admissions of 9,584 patients with diabetes, finding that ethnicity (black and Asian), older age (≥75 years), type of admission (emergency), insulin and sulfonylurea use could all predict the occurrence of hypoglycemia in hospitalized patients. A study of 9,665 patients with diabetes using modeling and validation revealed that older age, nasogastric tube or gastrostomy tube feeding, a higher Charlson comorbidity index, admission for vomiting, combined acute renal failure, and insulin use were risk factors for inpatient hypoglycemia (98). In addition to age and insulin, emergency department history in the previous 6 months, oral hypoglycemic agent use without inducing hypoglycemia, and severe CKD were all associated with inpatient hypoglycemia (74). Furthermore, a study of 21,840 adult patients with diabetes found that male patients were more prone to hypoglycemia on the first day of hospitalization, while glucose variability, low body weight, low creatinine clearance, insulin and sulfonylurea use were common risk factors for hypoglycemia (66). Frail patients treated with insulin or with insufficient nocturnal glucose monitoring were predisposed to inpatient hypoglycemia (81). Low serum albumin levels, in addition to the aforementioned risk factors, were also identified (86, 87) (Table 8).

**Table 8:** Clinical predictors of inpatient hypoglycemia.

**Category of predictors**	**Predictors**
Demographics	• Advanced age (59, 74, 98)
	• Ethnicity (Black and Asian) (59) • Low body weight (66)
GLA	• Insulin (35, 59, 60, 66, 74, 98, 101) • SU (59, 66, 101)
Other	• CV of blood glucose (35, 66) • Any previous hypoglycemia (35, 60) • Hospital day number (35) • Nadir blood glucose level admission (35) • Type of admission (Emergency) (59) • Feeding with nasogastric or percutaneous gastrostomy tube (98) • High Charlson comorbidity score (98, 101) • Vomiting as a cause of admission (98) • Acute renal failure (98), severe chronic kidney disease (60, 74) • Emergency department visit 6 months prior (74) • Number of hospitalization days (60, 101) • Low creatinine clearance (66) • Frailty (81) • Low serum albumin (86, 87)

GLA, glucose-lowering agents; SU, sulfonylurea.

The use of ML approaches in inpatient hypoglycemia prediction was summarized in Table 9. It can be seen that the most popular approaches for inpatient hypoglycemia prediction in our reviewed articles were LR and XGBoost. A study that used ML to mine electronic health records data from 17,658 patients with diabetes revealed the superiority of ML: when compared to the traditional LR method (AUC = 0.75), the AUC of the XGBoost model could reach 0.96 in identifying hypoglycemic events (36). However, considering of the sample size and model validation, the study carried out by Mathioudakis et al. (35) using stochastic gradient boosting (SGB) achieved the best performance in a narrow PH (24 h after each glucose measurement).

**Table 9:** Machine learning approaches for inpatient hypoglycemia prediction and best results performed.

**References**	**Participants, type**	**Inputs**	**PH**	**Algorithm**	**Outcome**	**Validation**	**Performance**
Stuart et al. (59)	9,584, T1D + T2D	Demographics, Lab data, comorbidity score, GLA, previous type of admission	Hospital stay	LR	Hypoglycemia < 72 mg/dL	Bootstrapping	AUC = 0.733
Ena et al. (60)	1,400, DM	Demographics, Lab data, comorbidities, GLA	Hospital stay	LR	Hypoglycemia < 70 mg/dL	External	Validation: AUC = 0.71
Winterstein et al. (66)	21,840, DM	SMBG, demographics, GLA, Lab data, oral intake related, service location related, comorbidities	24 h	LR	Hypoglycemia < 50 mg/dL not followed by glucose value > 80 mg/dL within 10 min	Bootstrapping	On day 3–5: c-statistic = 0.877
Mathioudakis et al. (67)	19,262, DM	Demographics, diagnoses, insulin, comorbidities, Lab data, medications, diet order, steroid use, BG readings	24 h	LR	Hypoglycemia ≤ 70 mg/dL, < 54 mg/dL	Internal	≤ 70 mg/dL: c-statistic = 0.77; < 54 mg/dL: c-statistic = 0.80
Shah et al. (74)	585, DM	Demographics, previous HYPO events, Lab data, GLA, CKD status	Hospital stay	LR	Hypoglycemia ≤ 70 mg/dL	External	Validation: c-statistic = 0.642, Se = 77.0%, Sp = 28.0%
Hu et al. (84)	257, T2D	Demographics, Lab data, COM, comorbidities	Hospital stay	LR	Hypoglycemia ≤ 70 mg/dL	Bootstrapping	AUC = 0.664
Ruan et al. (36)	17,658, DM	Demographics, medications, vital signs, Lab data, hospitalization procedure, previous HYPO events	Hospital stay	XGBoost	Hypoglycemia < 72 mg/dL, 54 mg/dL	10-fold cross validation	AUC_72/54_ = 0.96/0.96, Se_72/54_ = 70.0%/67.0%, PPV_72/54_ = 88%/ 97%
Elbaz et al. (98)	9,665, DM	Demographics, smoking, use of alcohol, comorbidities, Lab data, GLA, other medication	First week of admission	LR	Hypoglycemia ≤ 70 mg/dL	Internal, external	Validation set 1/2: AUC = 0.72/0.71
Kyi et al. (101)	594, T2D	Demographics, GLA, hospital treatment factors, Lab data, comorbidities, observed-days	Hospital stay	LR	At least 2 days with capillary glucose < 72 mg/dL	Internal	AUC = 0.806, Se = 84.0%, Sp = 66.0%, PPV = 53.0%
Mathioudakis et al. (35)	35,147, DM	Demographics, diagnoses, hospitalization procedures, Lab data, medications, BG readings, insulin	24 h after each glucose measurement	SGB	Hypoglycemia ≤ 70 mg/dL	Internal, external	Internal validation: c-statistic = 0.90; external validation: c-statistic: 0.86–0.88
Han et al. (107)	1,410, T2D	SMBG, demographics, medications, glycemic variability, Lab data	Perioperative period	LR	Hypoglycemia < 70 mg/dL	Bootstrapping	AUC = 0.715
Witte et al. (108)	38,250, DM	Demographics, medications, Lab data	7 h	XGBoost	Hypoglycemia < 70 mg/dL	5-fold cross validation	Se = 59.0%. Sp = 98.8%, PPV = 71.8%
Yang et al. (109)	29,843, T2D	Demographics, medications, Lab data	Hospital stay	XGBoost	Hypoglycemia < 70 mg/dL	10-fold cross validation	AUC = 0.822, Acc = 0.93
Wright et al. (111)	6,279, DM	Demographics, Lab data, comorbidities, glucose results, medications, hospitalization orders	24 h	LR	Hypoglycemia < 70 mg/dL within 24 h after insulin use	10-fold cross validation	AUC = 0.81, Se = 44.0%;

T1D, type 1 diabetes; T2D, type 2 diabetes; DM, diabetes; Lab, laboratory; GLA, glucose-lowering agents; SMBG, self-monitoring of blood glucose; INS, insulin; HYPO, hypoglycemia; CKD, chronic kidney disease; COM, diabetic complications.

### 3.6. Other hypoglycemia predictions

Prediction of postprandial and exercise-related hypoglycemia was also included in our review. Oviedo et al. (73) used a SVM algorithm to predict postprandial hypoglycemia within 240 minutes after meal in 10 T1D patients receiving sensor-augmented pump (SAP) therapy, and they found that the sensitivity and specificity for prediction of mild hypoglycemia < 70 mg/dL were 71.0 and 79.0%, respectively, and those for severe hypoglycemia < 54 mg/dL were 77.0 and 81.0%, respectively. Seo et al. (80) compared four ML models and discovered that the RF model had 89.6% sensitivity and 91.3% specificity in predicting hypoglycemic events after 30 min of meal absorption (Table 10).

**Table 10:** Machine learning approaches for other hypoglycemia prediction and best results performed.

**References**	**Participants, type**	**Inputs**	**PH**	**Algorithm**	**Threshold**	**Validation**	**Performance**
Postprandial hypoglycemia							
Oviedo et al. (115)	10, T1D	CGM, INS, CHO, blood glucose level at mealtime	6 h	NB, AdaBoost, SVM, ANN	< 70 mg/dL, < 54 mg/dL	5-fold cross validation	< 70 mg/dL: Se = 49.0%, Sp = 74.0%; < 54 mg/dL: Se = 51.0%, Sp = 74.0%
Oviedo et al. (66)	10, T1D	CGM, INS, CHO	6 h	SVC	< 70 mg/dL, < 54 mg/dL	5-fold cross validation	< 70 mg/dL: Se = 71.0%, Sp = 79.0%; < 54 mg/dL: Se = 77.0%, Sp = 81.0%
Seo et al. (81)	104, T1D + T2D	CGM	30 min	RF	≤ 70 mg/dL	5-fold cross validation	Se = 89.6%, Sp = 91.3%
Exercise-related hypoglycemia							
Reddy et al. (86)	43, T1D	Demographics, PA, glucose, hormone features	During exercise	DT, RF	< 70 mg/dL	10-fold cross validation	Acc = 86.67%, Se = 86.21%, Sp = 86.89%
Tyler et al. (109)	20, T1D	CGM/SMBG data, CGM indices, INS, CHO, HR, MET, age, height, weight	During aerobic exercise (4 h)	LR	< 70 mg/dL	Hold-out, 20-fold cross validation	Population model: Se/Sp = 73/76%, Acc = 75% (Hold-out set); Personalized model: Se/Sp = 73/90%, Acc = 84% (Hold-out set)

T1D, type 1 diabetes; T2D, type 2 diabetes; CGM, continuous glucose monitoring; SMBG, self-monitoring of blood glucose; INS, insulin; CHO, carbohydrate intake; PA, physical activity; HR, heart rate; MET, metabolic expenditure; NB, naïve Bayes.

Meanwhile, our research focused on exercise-related hypoglycemia. Prevention of hypoglycemia during exercise is a major challenge in diabetes. Providing predictions of glycemic changes during and following exercise can help people with diabetes avoid hypoglycemia. Reddy et al. (77) pointed out the most important features in the RF model for exercise-related hypoglycemia prediction: an average heart rate above 121 beats/min in the first 5 min of exercise, an increase in energy expenditure and a blood glucose value below 182 mg/dL at the beginning of exercise tended to increase the likelihood of hypoglycemia. Another unique dataset representing 320 days and 50,000 + time points of glycemic measurements collecting in adults with T1D who participated in a 4-arm crossover study evaluating insulin-pump therapies was used to develop adaptive, personalized ML algorithms to predict exercise-related glucose changes (115). Their personalized algorithms based on LR algorithm achieved high accuracy (84%) and specificity (90%) in predicting hypoglycemia during and following 4-h exercise sessions.

## 4. Conclusions

Hypoglycemia is a huge obstacle to achieving optimal glucose control in patients with diabetes, posing a great challenge to the healthcare system while potentially harming the patient's cardiovascular system and brain. Prediction of hypoglycemia is critical in clinical practice. In this study, we comprehensively reviewed the literature on hypoglycemic risk factors or hypoglycemic prediction that have been published in recent years and elaborated on the research progress in the prediction of various types of hypoglycemic events that are most concerned in clinical practice from various perspectives. Before the explosion of machine learning for hypoglycemia prediction, simple prediction based on large sample-sized clinical data proliferated due to clinical need over the last decade. We concluded the summary table of risk factors for different types of hypoglycemic events by extracting hypoglycemic risk factors from various studies. As a result, we discovered that age, insulin dose, sulfonylurea use, prior history of hypoglycemia, and combined CKD were the main risk factors for hypoglycemic episodes in clinical practice, but that under certain conditions, advanced age or younger age, different levels of HbA1c, and sulfonylurea use had different effects on hypoglycemia. This necessitates clinicians to assess the patient's condition before making an accurate judgment and decision.

The implementation of CGM systems, insulin pumps, and AP in clinical practice provides technical support for the prevention of hypoglycemic events. With the booming field of ML, hypoglycemic prediction models incorporating several other major factors affecting blood glucose such as insulin dose, carbohydrate intake, and physical activity have sprung up based on the massive amount of blood glucose information carried by CGM. However, the PH of current studies is < 240 min. Furthermore, the predictive performance of hypoglycemia varied with sample size, training data type, and ML method used. Furthermore, any predictive model and prevention strategy must be validated in a variety of clinical settings to determine whether there will be subsequent hypoglycemia reductions and improvements in clinical outcomes. In conclusion, while there have been some promising results in the field of hypoglycemia prediction, there is still much room for improvement.

Hypoglycemia in patients with diabetes is influenced by multiple factors, such as insulin administration, carbohydrate intake, physical activity, previous blood glucose readings, stress, age, BMI, duration of diabetes, pancreatic islet function, comorbidities, alcohol consumption, and smoking. To more accurately estimate the risk of hypoglycemia, an ideal predictive model for hypoglycemia should consider all pertinent confounding factors jointly. In this review, demographics data and CGM readings were the main types of model inputs used. CGM glucose values, for instance, were the most frequently used feature when performing real-time and nocturnal hypoglycemia predictions. Systems using ML can be trained to forecast future hypoglycemic events based on a person's historical glucose levels. The majority of these models used time series prediction techniques with glucose data that contains precise timestamps corresponding to real glucose values. Additionally, some models also considered the effects of insulin, carbohydrate intake, and physical activity on hypoglycemia. In our study, patients manually recorded their dietary information using paper diaries or electronic diaries. This information included the frequency and timing of each meal and was typically estimated as carbohydrate (grams). Nevertheless, some researchers have attempted to automate the process of dietary data recording. In most cases, ML model integration was done directly using carbohydrate amounts (in grams), figuring out how many calories were in food, or using compartmental models to predict how much glucose is absorbed from the gut into the blood. Besides, the type, quantity, and intensity of physical activity, as well as its duration, all affect hypoglycemia differently. Data on physical activity can be gathered manually or automatically by wearable devices. A wide range of physical activity data, including the intensity of activity, the total energy expended over a specific period of time, a standard table of caloric use during exercise, task metabolic equivalent (MET) were taken into consideration. According to the current results, the most useful features are still the information carried by CGM, such as the original blood glucose value and hypoglycemia-related CGM parameters. When compared to studies that only used CGM data as inputs, the contribution of features (meal, insulin, and exercise) other than CGM glucose data was lower but not insignificant. In contrast to real-time hypoglycemia prediction models, which primarily relied on CGM blood glucose readings, MH/SH and inpatient hypoglycemia predictions were primarily based on the occurrence of hypoglycemic events collected from clinical datasets, and the included parameters were mostly demographics, insulin use, HbA1c or fasting blood glucose levels and previous hypoglycemic events. For such models, the most useful characteristics were those that were clinically closely related to the occurrence of hypoglycemia such as age, insulin, BMI, renal function, previous hypoglycemic events, and comorbidities.

The most effective algorithm in this area is still up for debate, despite the fact that many ML techniques have been widely used for hypoglycemia prediction over the past 10 years. As was already mentioned, the study population, PH, outcome definitions, modeling techniques, and model validation strategies all affect the model performance. Accordingly, it is essential to make sure that all conditions are comparable in order to accurately determine which algorithm outperforms for forecasting a particular hypoglycemic event. The study population of almost every study, according to the literature we reviewed for this study, is unique. Additionally, the inputs used for model development vary from study to study, which makes the horizontal comparison even more challenging. Although comparing sample size and use of external validation under the same PH and outcome under a specific hypoglycemia prediction scenario can yield a relatively well-behaved algorithm, this comparison is partially empirical and lacks direct comparisons of the performance metrics. In light of the present findings of this review, it is challenging to directly compare algorithms.

With the availability of large amounts of clinical data and growing awareness of big data analysis tools, more and more accurate hypoglycemia prediction models can be developed and tested. Future research should concentrate on the discovery of novel algorithms or models to develop more medical devices or decision support systems to prevent various types of hypoglycemic events and other adverse outcomes. Clinical trials will be required before application to assess the economic efficacy and long-term benefits to patients with diabetes.

## 5. Future directions

The generation of automated and continuous personal data has become possible with the proliferation of commercially available CGM, wearable, and other glucose collection devices for self-monitoring, opening up opportunities for better training ML models with more detailed data. Although integrating CGM with clinical data may improve model performance, widespread implementation of CGM devices in patients with diabetes remains an unsolved problem due to financial and human factor considerations (staff training, time, resources, and other physiological measurement tools that most patients do not have). Furthermore, despite increased research on ML-based prediction models for hypoglycemia over the last decade, achieving a generic model with accurate predictive efficacy under real-world conditions remains difficult due to the complexity of blood glucose dynamics. From the literature reviewed in this paper, most of the study samples came from retrospective datasets and were internally validated only. Thus, there are still great uncertainties in the model's accuracy and generalizability. Although advanced ML methods have been used to address these issues, the majority have yet to be invoked and tested in real-world situations. Future prospective external validation studies are urgently needed to confirm whether these models improve glycemic outcomes.

## Data availability statement

The original contributions presented in the study are included in the article/Supplementary material, further inquiries can be directed to the corresponding author.

## Author contributions

ZZ and LY conceived and designed the analysis. LZ collected the data, performed the analysis, and wrote the paper. All authors contributed to the article and approved the submitted version.
