# Supplementary material for: Data-based modeling for hypoglycemia prediction: Importance, trends, and implications for clinical practice
Source: Front Public Health. 2023 Jan 26;11:1044059. doi: 10.3389/fpubh.2023.1044059 (PMC9910805; doi:10.3389/fpubh.2023.1044059)
Supplement: Supplementary file 1 [file Table_1.docx]

# Supplementary materials

Table S1. Prediction metrics used in selected studies.

| Metric | Definition |
| --- | --- |
| Correlation coefficient | The strength of the relationship between the relative movements of two variables. |
| Odds ratio (OR) | Quantifies the strength of the association between two events. |
| Hazard ratio (HR) | The probability of an event in a treatment group relative to the control group probability over a unit of time. |
| Area under the curve (AUC) | Measures the entire two-dimensional area underneath the entire ROC curve. |
| Sensitivity (Se) | Measures the proportion of actual positives that are correctly identified. |
| Specificity (Sp) | Measures the proportion of actual negatives that are correctly identified. |
| Positive predictive value (PPV) | Proportions of positive results in statistics and diagnostic tests that are true positive results. |
| False positive rate (FPR) | Ratio between the number of negative events wrongly categorized as positive (false positives) and the total number of actual negative events. |
| C-statistic | Measure of goodness of fit for binary outcomes in a logistic regression model. |
